# *Plasmodium*-specific atypical memory B cells are short-lived activated B cells

**DOI:** 10.7554/eLife.39800

**Published:** 2018-11-02

**Authors:** Damián Pérez-Mazliah, Peter J Gardner, Edina Schweighoffer, Sarah McLaughlin, Caroline Hosking, Irene Tumwine, Randall S Davis, Alexandre J Potocnik, Victor LJ Tybulewicz, Jean Langhorne

**Affiliations:** 1 The Francis Crick InstituteLondonUnited Kingdom; 2 MRC National Institute for Medical ResearchLondonUnited Kingdom; 3 Department of MedicineUniversity of Alabama at BirminghamBirminghamUnited States; 4 Department of MicrobiologyUniversity of Alabama at BirminghamBirminghamUnited States; 5 Department of Biochemistry and Molecular GeneticsUniversity of Alabama at BirminghamBirminghamUnited States; 6 School of Biological SciencesThe University of EdinburghEdinburghUnited Kingdom; Ragon Institute of MGH, MIT and HarvardUnited States; The Rockefeller UniversityUnited States

**Keywords:** atypical memory B cells, age-associated B cells, Plasmodium, malaria, mosquito, FCRL5, Mouse

## Abstract

A subset of atypical memory B cells accumulates in malaria and several infections, autoimmune disorders and aging in both humans and mice. It has been suggested these cells are exhausted long-lived memory B cells, and their accumulation may contribute to poor acquisition of long-lasting immunity to certain chronic infections, such as malaria and HIV. Here, we generated an immunoglobulin heavy chain knock-in mouse with a BCR that recognizes MSP1 of the rodent malaria parasite, *Plasmodium chabaudi*. In combination with a mosquito-initiated *P. chabaudi* infection, we show that *Plasmodium*-specific atypical memory B cells are short-lived and disappear upon natural resolution of chronic infection. These cells show features of activation, proliferation, DNA replication, and plasmablasts. Our data demonstrate that *Plasmodium*-specific atypical memory B cells are not a subset of long-lived memory B cells, but rather short-lived activated cells, and part of a physiologic ongoing B-cell response.

## Introduction

Atypical memory B cells (AMB) are an unusual B-cell subset detected in both mouse models and humans in the context of certain infections and autoimmune disorders, including HIV, HCV, tuberculosis, malaria, rheumatoid arthritis and systemic lupus erythematosus, and accumulated with age (Knox et al., 2017b; Naradikian et al., 2016a; Portugal et al., 2017; Rubtsov et al., 2017). In the context of infections, AMB were first described in HIV-viremic subjects, and termed tissue-like memory B cells, due to their similarity to an FCRL4-expressing memory B-cell subset found in human tonsillar tissues (Ehrhardt et al., 2005; Moir et al., 2008). In addition to FCRL4, these cells express relatively high levels of other potentially inhibitory receptors including CD22, CD85j, CD85k, LAIR-1, CD72, and PD-1, and show a profile of trafficking receptors including expression of CD11b, CD11c and CXCR3, consistent with migration to inflamed tissues. They are antigen-experienced class-switched B cells, which lack the expression of CD21 and the hallmark human memory B-cell marker CD27. Further studies demonstrated the expression of the transcription factor T-bet and the cytokine IFNγ by these cells, also characteristic of Th1 CD4^+^ T cells (Knox et al., 2017b; Obeng-Adjei et al., 2017; Portugal et al., 2017). Due to their poor functional capacity upon in vitro re-stimulation with BCR ligands, AMB were characterized as dysfunctional B cells, and increased frequencies of these cells was proposed to be a consequence of B-cell exhaustion driven by chronic inflammation and stimulation, drawing parallels with T-cell exhaustion during chronic viral infections (Moir et al., 2008; Portugal et al., 2015; Sullivan et al., 2015). It has been hypothesized that expansion of AMB might contribute to the mechanisms driving autoimmune disorders and deficiencies in acquisition of immunity to chronic infections. However, due to lack of good tools and animal models to analyze antigen-specific atypical B cells in greater depth, many of these concepts remain speculative.

Several studies suggest that AMB might contribute to poor acquisition of long-term immunity to *Plasmodium* infection (Illingworth et al., 2013; Portugal et al., 2015; Sullivan et al., 2015; Sullivan et al., 2016; Weiss et al., 2011; Weiss et al., 2009; Weiss et al., 2010). Indeed, some studies demonstrated that in the absence of constant re-exposure, *Plasmodium*-specific serum antibody levels rapidly wane, and full protection from clinical symptoms is lost, suggesting that B-cell memory is functionally impaired (Portugal et al., 2013). However, others have reported long-lasting maintenance of *Plasmodium*-specific antibodies and/or memory B cells in settings of differing malaria endemicity, and similar responses are also observed in mouse malaria models (Dorfman et al., 2005; Ndungu et al., 2009; Ndungu et al., 2013; Ndungu et al., 2012; Wipasa et al., 2010). Moreover, it has been shown that BCRs cloned from *P. falciparum*-specific AMB from malaria-exposed adults encode *P. falciparum*-specific IgG antibodies, which could contribute to *P. falciparum*-specific IgG antibodies in serum (Muellenbeck et al., 2013). These authors proposed that *P. falciparum*-specific AMB do not prevent, but rather contribute to the control of *Plasmodium* infection. These apparently contradictory results may reflect the fact that some studies were performed on the general peripheral blood B-cell pool and others focused on *Plasmodium*-specific B cells. In determining a role for these cells in a chronic infection it would be important to follow antigen-specific responses and to distinguish these from non-specific polyclonal B cell activation.

The study of the development of AMB is challenging and requires suitable mouse models, which allow for identification and isolation of antigen-specific B cells that exist often at very low frequency. Here, we generated a knock-in transgenic mouse with a high frequency of B cells specific to the 21 kDa C-terminal fragment of *Plasmodium chabaudi* Merozoite Surface Protein 1 (MSP1_21_), to investigate memory B cells generated following mosquito-transmission of the rodent malaria, *P. chabaudi*. We identified a CD11b^+^CD11c^+^FCRL5^hi^ subset of MSP1_21_-specific B cells during the chronic infection with phenotypical and transcriptional features strikingly similar to those of human AMB. These AMB disappeared as the infection progressed, leaving a CD11b^—^CD11c^—^FCRL5^hi^ MSP1_21_-specific B-cell compartment with characteristics of long-lived classical memory B cells (B_mem_) after the resolution of the infection. These short-lived MSP1_21_-specific AMB were also generated in response to immunization, suggesting they may be a normal but transient component of a B-cell response to antigen. In this chronic *P. chabaudi* infection, it appears that AMB require ongoing antigenic stimulation driven by the sub-patent infection to persist, and do not represent a true long-lived ‘memory’ B cell subset. Moreover, we show that generation of *Plasmodium*-specific AMB does not prevent the generation of *Plasmodium*-specific B_mem_, and does not prevent resolution of the infection.

## Results

### Generation of an immunoglobulin heavy chain knock-in transgenic mouse model to study *Plasmodium*-specific B cell responses

To study Plasmodium-specific B cell responses in a rodent malaria model, we generated an *Igh*^NIMP23/+^ mouse strain on the C57BL/6J background (Materials and methods and Figure 1—figure supplement 1).

**Figure 1.**
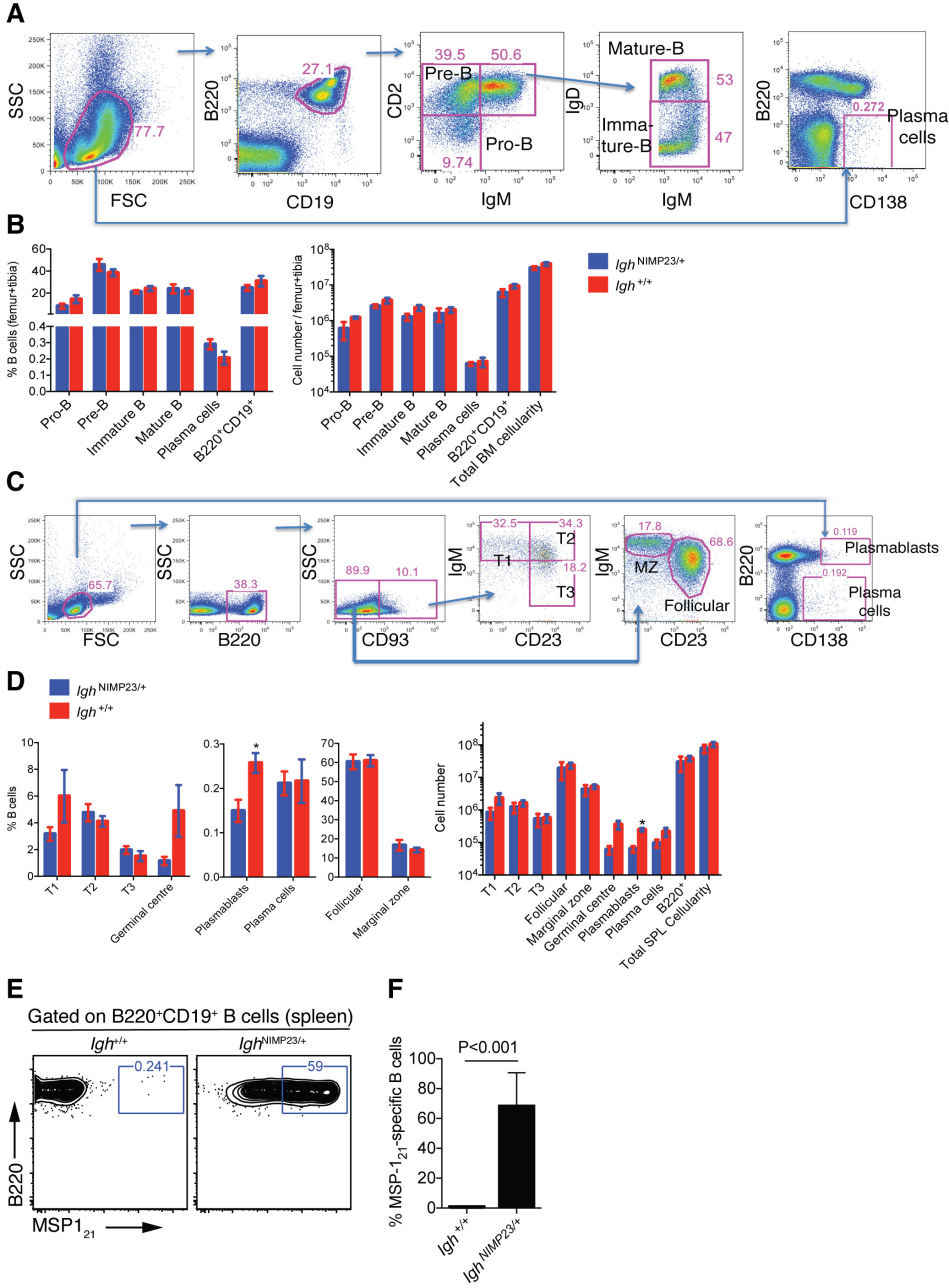
Analysis of total bone marrow and splenic B-cell populations in *Igh*^NIMP23/+^ and *Igh*^+/+^littermates. (**A**) Flow cytometry gating strategy to identify different B-cell populations in bone marrow of *Igh*^NIMP23/+^ mice. Arrows indicate flow of analysis. The same strategy was used for *Igh*^+/+^ littermates. (**B**) Percentages and numbers of different B-cell populations in bone marrow of *Igh*^NIMP23/+^ and *Igh*^+/+^ littermates as defined in (**A**). (**C**) Flow cytometry gating strategy to identify different B-cell populations in spleen of *Igh*^NIMP23/+^ mice. (**D**) Percentages and numbers of different B-cell populations in spleen of *Igh*^NIMP23/+^ and *Igh*^+/+^ littermates as defined in (**C**). Data are representative of two independent experiments with four mice per group. (**E**) Flow cytometry analysis of B cells obtained from spleen of *Igh*^+/+^ (left) and *Igh*^NIMP23/+^ (right) mice stained with anti-B220 and CD19 antibodies in combination with an MSP1_21_ fluorescent probe. The gates show the frequency of B cells specific to MSP1_21_. (**F**) Frequencies of MSP1_21_-specific splenic B cells in *Igh*^NIMP23/+^ and wild-type *Igh*^+/+^ littermate controls (Mann Whitney U test). Data pooled from two independent experiments with 3–5 mice per group. Mann Whitney U test. *p<0.05. Error bars are SEM.

The *Igh*^NIMP23/+^ mice were healthy, with no unusual behavioral or physical characteristics. There were no alterations in total cellularity, pro-B, pre-B, immature B, mature B, total B220^+^CD19^+^ B cells, and plasma cells in the bone marrow (Figure 1A–B), and no alterations in number of T1, T2, T3, follicular, marginal zone, germinal center B cells, plasmablasts, plasma cells, and total cellularity in the spleen of *Igh*^NIMP23/+^ mice (Figure 1C–D) (Sen et al., 1990; Young et al., 1994). Importantly, The *Igh*^NIMP23/+^ mice had a greatly increased frequency of B cells specific for MSP1_21_ (approximately 60% of the total B-cell compartment), as demonstrated by flow cytometry analysis of splenocytes with a MSP1_21_ fluorescent probe consisting of biotinylated recombinant MSP1_21_ loaded on streptavidin-PE (Figure 1E–F). Thus, in this model, a recombinant light chain is not required to bring about specificity. This suggest that most endogenous light chains will pair with the NIMP23 heavy chain to generate a BCR with detectable binding to MSP1_21_.

**Figure 1—figure supplement 1.**
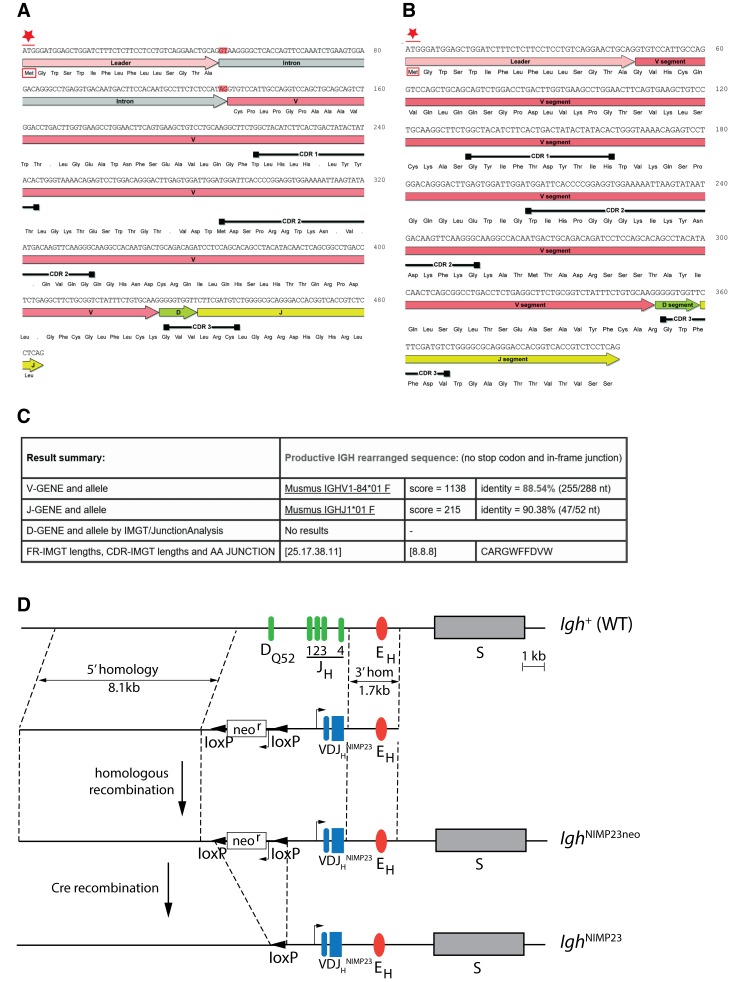
Generation of *Igh*^NIMP23/+^knock in mice. Annotated DNA and corresponding amino acid sequence of VDJ_H_^NIMP23^ obtained from (**A**) gDNA including the Leader-V intron and (**B**) cDNA, of the NIMP23 hybridoma: start Methionine (Met) indicated by red star, intron splice donor and acceptor sites highlighted in red, dots indicate STOP codons, black bars indicate predicted complementarity determining regions according to the Kabat database (Johnson and Wu, 2001) (**C**) IMGT/V-Quest mouse Ig database (Lefranc et al., 1999) comparative analysis result summary of the gDNA derived VDJ_H_^NIMP23^ sequence reveals the identity of the closest matching endogenous V and J genes (**D**) Schematic representation of (*Igh*^+^) Endogenous IgH locus showing the 4 JH segments, the DQ52 element, the Igh intronic enhancer (E_H_), the switch region for the constant µ gene (**S**), and (below) targeting construct indicating 5’ and 3’ homology arms and the inverted loxP-neo^r^-loxP cassette and rearranged VDJ_H_^NIMP23^ variable heavy chain region gene of the NIMP23 hybridoma, replacing DQ52 and all four JH segments of the endogenous IgH gene. *Igh*^NIMP23neo^: Targeted *Igh* locus after homologous recombination. *Igh*^NIMP23^: Final allele after Cre-mediated removal of neo^r^.

**Figure 1—figure supplement 2.**
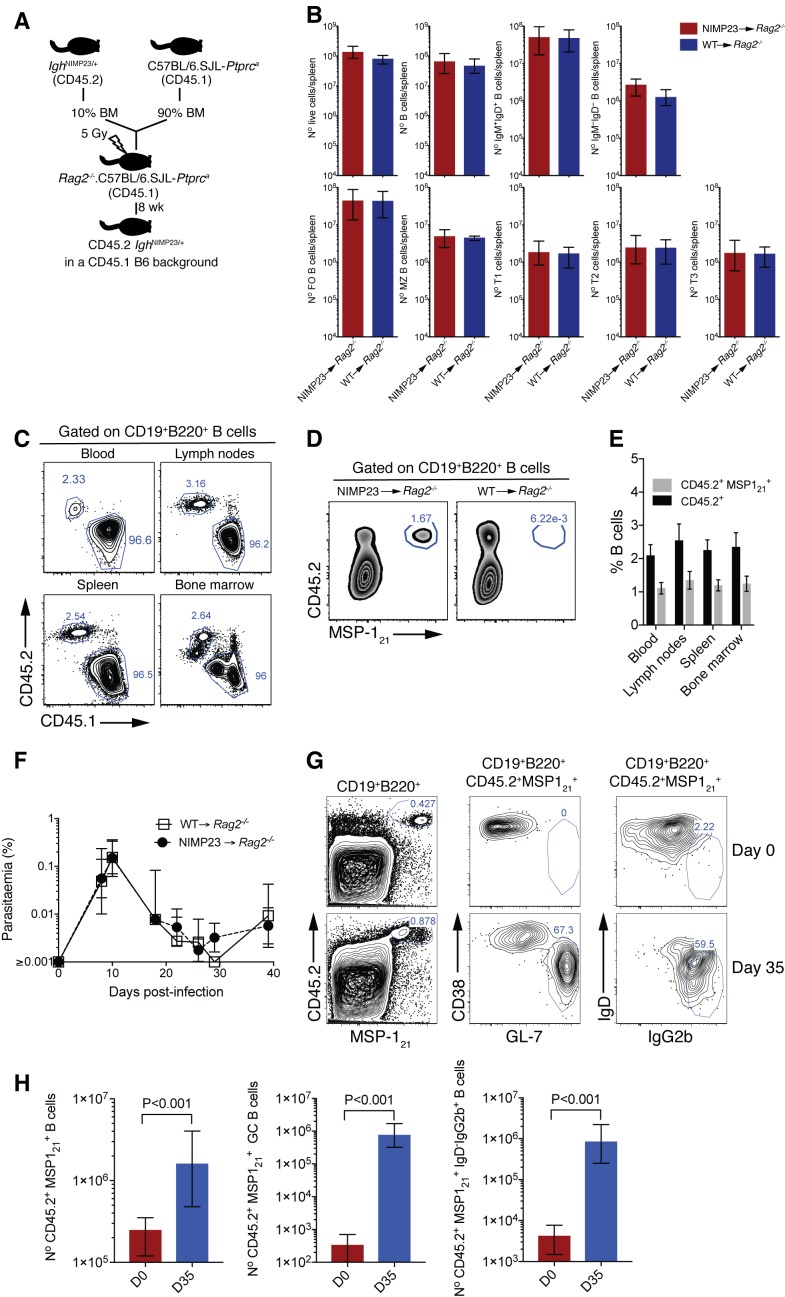
Generation of mixed bone marrow chimera model with reduced precursor frequency of *Igh*^NIMP23*/+ *^B cells to study MSP1_21_-specific B cell responses during *P. chabaudi* infection. (**A**) Experimental strategy to generate mixed bone marrow chimeric mice. (**B**) Numbers of different splenic B-cell populations defined by flow cytometry in *Rag2^-/-^*.C57BL/6.SJL-*Ptprc^a^* mice reconstituted with a mixture of *Igh*^NIMP23/+^ and C57BL/6.SJL-*Ptprc^a^* bone marrow in a 10:90 ratio (NIMP23→ *Rag2^-/-^*), and control mice reconstituted with C57BL/6.SJL-*Ptprc^a^* bone marrow (WT→ *Rag2^-/-^*). Mann Whitney U test. Error bars are SEM. Data are representative of two independent experiments with five mice per group. (**C**) Flow cytometry of B cells obtained from different tissues of NIMP23→*Rag2^-/-^* chimeric mice. Gates show frequencies of CD45.1^+^CD45.2^-^ and CD45.1^-^CD45.2^+^ (**D**) Flow cytometry of B cells obtained from spleen of NIMP23→*Rag2^-/-^* and WT→*Rag2^-/-^* control chimeric mice. Gates show frequencies of MSP1_21_-specific B cells as determined by CD45.2 vs MSP1_21_ staining. (**E**) Frequencies of CD45.1^-^CD45.2^+^ (black) and CD45.2^+^MSP1_21_^+^ (grey) B cells as gated in C and D, obtained from different organs of NIMP23→*Rag2^-/-^* chimeric mice. (**F**) Blood-stage *P. chabaudi* parasitemia following mosquito transmission in NIMP23→*Rag2^-/-^* and WT→*Rag2^-/-^* control chimeric mice. (**G**) Flow cytometry data showing frequencies of MSP1_21_-specific GC (CD38^lo^GL-7^hi^) and class-switched (IgD^—^IgG2b^hi^) B cells in the spleen of NIMP23→*Rag2^-/-^* chimeric mice before infection (day 0) and at day 35 post-mosquito transmitted *P. chabaudi* infection. (**H**) Numbers of MSP1_21_-specific B cells, GC and class-switched B cells in the spleen of NIMP23→*Rag2^-/-^* chimeric mice as gated in B and E. Mann Whitney U test. Error bars are SEM. Data representative of two independent experiments with 3–7 mice per group.

### Increase in *Plasmodium*-specific B cells after mosquito transmission of *P. chabaudi*

To investigate B cells in *P. chabaudi* infections, which last several weeks, and to avoid potential problems with activation arising from very high frequencies of MSP1-specific B cells, we reduced the precursor frequency of MSP1_21_-specific B cells to match the natural level expected for antigen-specific B cells more closely, yet still readily detectable by flow cytometry. We generated mixed bone marrow (BM) chimeras by adoptively transferring a mixture of 10% bone marrow from either *Igh*^NIMP23/+^ or *Igh*^+/+^ mice (CD45.2^+^) together with 90% bone marrow from C57BL/6.SJL-*Ptprc^a^* mice (CD45.1^+^) into sub-lethally irradiated *Rag2^-/-^*.C57BL/6.SJL-*Ptprc^a^* mice (CD45.1^+^) to generate NIMP23→*Rag2^-/-^* and WT→*Rag2^-/-^* bone marrow chimeric mice respectively (Figure 1—figure supplement 2A–B). In both types of chimeras, 2–3% of the B cells were CD45.2^+^ and in NIMP23→*Rag2^-/-^* mice approximately 1–2% of the B cells were MSP1_21_-specific (Figure 1—figure supplement 2C–E). No MSP1_21_-specific B cells were detected in the control WT→*Rag2^-/-^* chimeras (Figure 1—figure supplement 2D).

Infection of C57BL/6J *wt* mice with *P. chabaudi* by mosquito bite gives rise to a short (48 hr) pre-erythrocytic infection, followed by an acute blood parasitemia peaking approximately 10d post-transmission. Thereafter, the infection is rapidly controlled, reaching very low parasitemias by 15d post-transmission, with a subsequent prolonged (~90 d), but low-level chronic infection before parasite elimination (Brugat et al., 2017; Spence et al., 2013). NIMP23→*Rag2^-/-^* mice infected with *P. chabaudi* by mosquito bite, showed a similar course of parasitemia to that of control WT→*Rag2^-/-^* mice (Figure 1—figure supplement 2F), and C57BL/6J *wt* mice (Brugat et al., 2017; Spence et al., 2013; Spence et al., 2012). Importantly, the MSP1_21_-specific *Igh*^NIMP23/+^ B cells (CD45.2^+^MSP1_21_^+^) in NIMP23→*Rag2^-/-^* chimeras showed a robust response to the infection, as demonstrated by a dramatic increase in the proportions and numbers of GL-7^+^CD38^lo^ germinal centers (GC) and IgG2b^+^IgD^—^ class-switched B cells in the spleen at 35 days post-infection (dpi) (Figure 1—figure supplement 2G–H).

Thus, we have generated a mouse model with detectable numbers of functional MSP1_21_-specific B cells capable of responding to *P. chabaudi* infection.

### Generation of *Plasmodium*-specific AMB after mosquito transmission of *P. chabaudi* infection

We investigated whether *Plasmodium*-specific AMB could be identified in mice during a blood-stage *P. chabaudi* infection. We selected a series of mouse homologues to human cell surface markers described on human AMB (Charles et al., 2011; Kardava et al., 2014; Kardava et al., 2011; Knox et al., 2017a; Li et al., 2016; Moir et al., 2008; Muellenbeck et al., 2013; Portugal et al., 2015; Russell Knode et al., 2017; Sullivan et al., 2015). Human AMB express CD11b, CD11c, Fc receptor-like (FCRL) 3–5, high levels of CD80, low levels of CD21, and are Ig class-switched. Mouse FCRL5 most closely resembles human FCRL3 and is the only mouse FCRL-family member which contains both ITIM and ITAM motifs in its cytoplasmic tail (Davis, 2007; Davis et al., 2004; Won et al., 2006; Zhu et al., 2013). Therefore, our flow cytometry panel for mouse AMB included antibodies against CD11b, CD11c, FCRL5, CD21, IgD, and also CD80 and CD273 which identify mouse B cells that are antigen-experienced and potentially memory cells (Anderson et al., 2007; Tomayko et al., 2010; Zuccarino-Catania et al., 2014).

**Figure 2.**
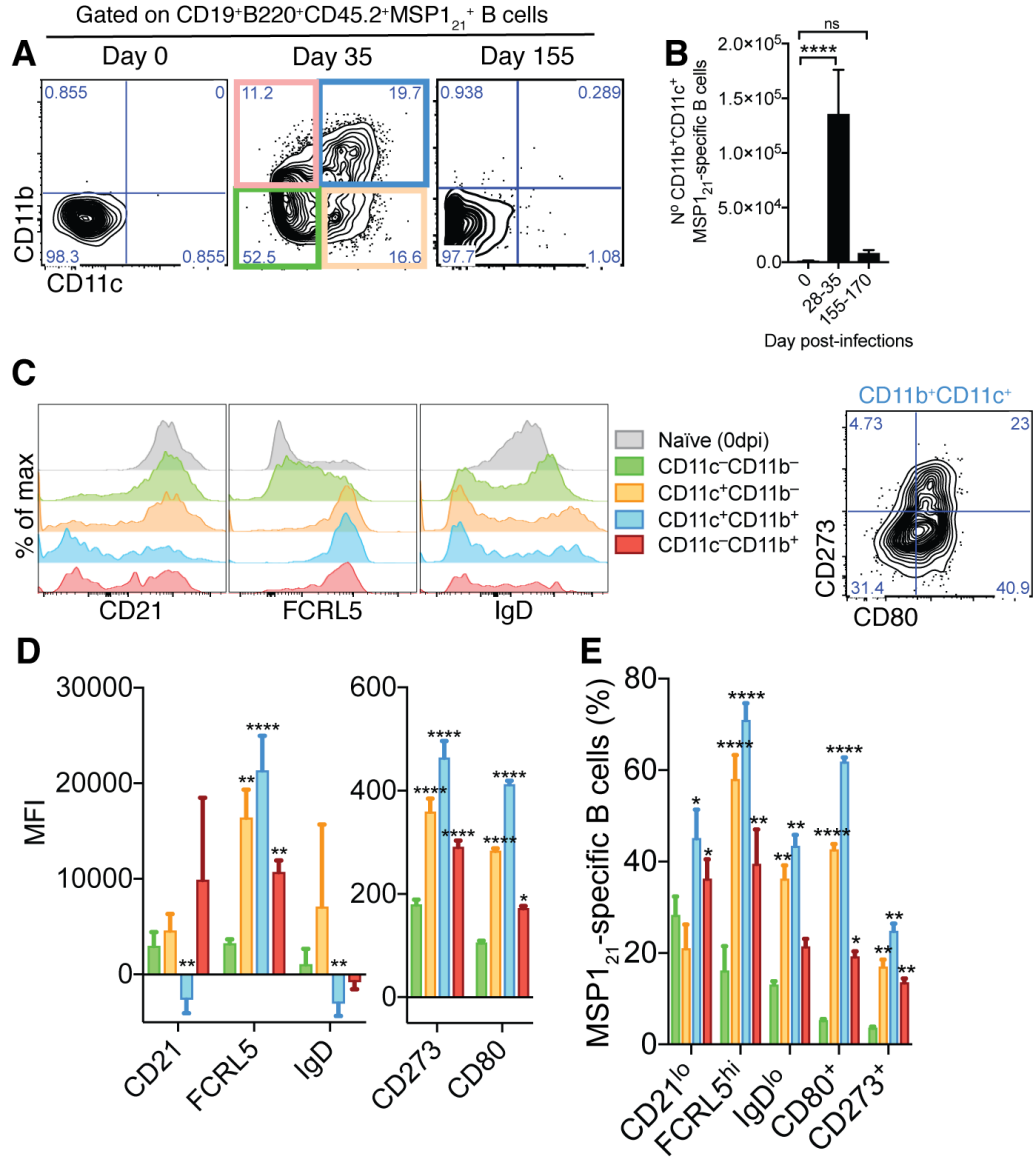
Generation of MSP1_21_-specific AMB in response to mosquito transmitted *P.chabaudi* infection. (**A**) Flow cytometry showing differential expression of CD11b and CD11c on splenic MSP1_21_-specific B cells from NIMP23→*Rag2^-/-^* chimeric mice before infection (day 0) and at 35 and 155dpi. (**B**) Numbers of splenic MSP1_21_-specific CD11b^+^CD11c^+^ AMB from NIMP23→*Rag2^-/-^* during the course of mosquito transmitted *P. chabaudi* infection. Kruskal-Wallis test vs day 0. ****, p<0.0001 (**C**) Flow cytometry showing expression of CD21/35, FCRL5, IgD, CD273 and CD80 on different subsets of splenic MSP1_21_-specific B cells from NIMP23→*Rag2^-/-^* chimeric mice defined based on CD11b and CD11c expression at 35dpi. (**D**) Geometric mean fluorescence intensity (MFI) of CD21/35, FCRL5, IgD, CD273 and CD80 expression on different subsets of splenic MSP1_21_-specific B cells from NIMP23→*Rag2^-/-^* chimeric mice defined based on CD11b and CD11c expression at 35dpi. (**E**) Frequencies of CD21/35, FCRL5, IgD, CD273 and CD80 positive cells among different subsets of splenic MSP1_21_-specific B cells from NIMP23→*Rag2^-/-^* chimeric mice defined based on CD11b and CD11c expression at 35dpi. Two-way ANOVA vs CD11b^-^CD11c^-^ subset. *p<0.05; **p<0.01, ***p<0.001; ****p<0.0001. Error bars are SEM. Data pooled from three independent experiments with 3–5 mice per group.

We detected an increased number of cells in a distinct CD11b^+^CD11c^+^ MSP1_21_-specific B-cell subset at 28-35dpi, in the chronic phase of *P. chabaudi* infection (Figure 2A–B). This subset showed several AMB characteristics, including high expression of FCRL5 and low expression of CD21 and IgD (Figure 2C–E). In addition, the CD11b^+^CD11c^+^ MSP1_21_-specific B-cell subset was enriched with cells expressing CD80 and CD273 (Figure 2C–E).

We then explored whether this CD11b^+^CD11c^+^ MSP1_21_-specific B cell subset was detected during the memory phase,that is after resolution of the infection. As it takes up to 90 days for a blood-stage *P. chabaudi* infection to be eliminated from C57BL/6J mice (Achtman et al., 2007; Spence et al., 2013), we measured these responses from 155dpi onwards. Unexpectedly, the numbers of CD11b^+^CD11c^+^ MSP1_21_-specific B cells were not significantly higher than background level (Figure 2A–B).

These data demonstrate that a mosquito-borne infection with *P. chabaudi* generates *Plasmodium*-specific B cells resembling human AMB. However, these cells do not persist and are not detected above background level after parasite clearance.

### Transcriptome analysis confirms the AMB nature of CD11b^+^CD11c^+^ MSP1_21_-specific B cells, and reveals a plasmablast-like signature for this subset

To gain a better understanding of the identity of the CD11b^+^CD11c^+^ *Plasmodium*-specific B cell subset, we isolated both CD11b^+^CD11c^+^ and CD11b^-^CD11c^-^ MSP1_21_-specific B cells from spleens of *P. chabaudi*-infected NIMP23→*Rag2^-/-^* mice (35dpi) (Figure 3—figure supplement 1), and MSP1_21_-specific B cells from the spleen of naïve NIMP23→*Rag2^-/-^* mice (Figure 2A), by flow cytometric sorting, and performed an mRNAseq transcriptional analysis on the three populations.

**Figure 3.**
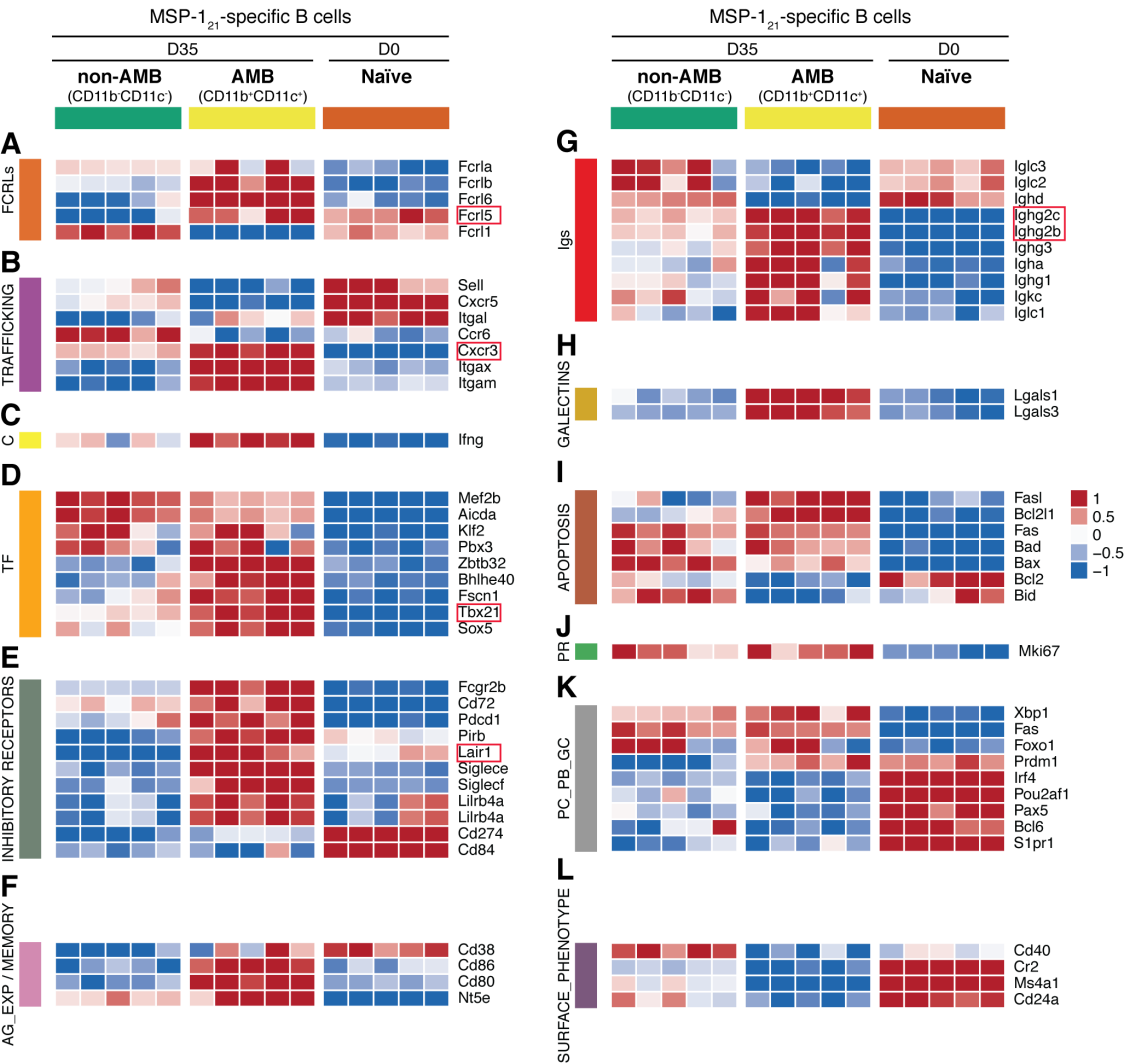
Transcriptome analysis of sorted splenic MSP1_21_-specific CD11b^+^CD11c^+^AMB. MSP1_21_-specific CD11b^+^CD11c^+^ (AMB) and CD11b^—^CD11c^—^ B cells were flow cytometry sorted from the spleen of NIMP23→*Rag2^-/-^* chimeric mice at 35dpi; MSP1_21_-specific B cells were flow cytometry sorted from the spleen of naïve NIMP23→*Rag2^-/-^*, and these three B cell populations were submitted to mRNAseq analysis. The heat maps display level of expression of selected individual genes, organized in functional clusters related to (**A**) Fc receptor like molecules, (**B**) cell trafficking, (**C**) cytokines, (**D**) transcription factors, (**E**) inhibitory receptors, (**F**) antigen experience/memory, (**G**) immunoglobulins, (**H**) galectins, (**I**) apoptosis, (**J**) proliferation, (**K**) plasma cells/plasmablasts/germinal centers, (**L**) surface markers. Each column corresponds to data from an individual mouse (n = 5 35 dpi, n = 5 0 dpi).

We then selected a large series of key genes previously shown to be either up or downregulated on human AMB (Supplementary file 1), and explored the expression of their mouse homologues on the three different B-cell subsets we sorted at day 35pi. The transcriptome of CD11b^+^CD11c^+^ MSP1_21_-specific B cells highly resembled that of human AMB. A series of hallmark genes upregulated in human AMB were also upregulated in CD11b^+^CD11c^+^ MSP1_21_-specific B cells, including IgG (*Ighg2c* and *Ighg2b*), *Cxcr3*, *Tbx21* (T-bet), *Lair1* and *Fcrl5* (Figure 3, genes in red boxes, and references in Supplementary file 1). In addition, the MSP1_21_-specific CD11b^+^CD11c^+^ B-cell subset showed upregulation of *Ifng*, *Aicda*, a large array of inhibitory receptors [including *Pd1*, *Cd72*, *Cd85k*, *Fcgr2b* (CD32b), S*iglece*], antigen-experienced/memory markers [*Cd80*, *Cd86*, *Nt5e* (CD73) and high *Cd38*], and additional class-switched immunoglobulins (i.e. *Igha*, *Ighg1* and *Ighg3*), all of which have been shown to be upregulated on human AMB (Figure 3C–G, references in Supplementary file 1). These cells also expressed Galectins (*Lgals1* and *Lgals3*), previously implicated in B-cell anergy (Clark et al., 2007) (Figure 3H), and displayed a pro-apoptotic program (e.g. high expression of *Fasl*, and low expression of *Bcl2*) (Figure 3I). Interestingly, in agreement with data on human AMB, MSP1_21_-specific CD11b^+^CD11c^+^ B cells showed upregulation of *Mki67* (Figure 3J), indicative of proliferation, and had characteristics of plasmablasts and/or plasma cells, including upregulation of *Cd138*, *Prdm1* (*Blimp1*) and *Xbp1*, and low expression of *Cxcr5*, *Pax5*, and *Bcl6* (Figure 3B and K). However, these cells showed low expression of *Irf4* and *S1p1*, suggesting that they may be in a pre-plasmablast or pre-migratory plasma-cell stage (Kabashima et al., 2006; Kallies et al., 2007) (Figure 3K). Finally, and similar to human AMB, CD11b^+^CD11c^+^ MSP1_21_-specific B cells showed low expression of *Cd40*, *Cr2* (CD21), *Ms4a1* (CD20), and *Cd24a* (Figure 3L).

**Figure 3—figure supplement 1.**
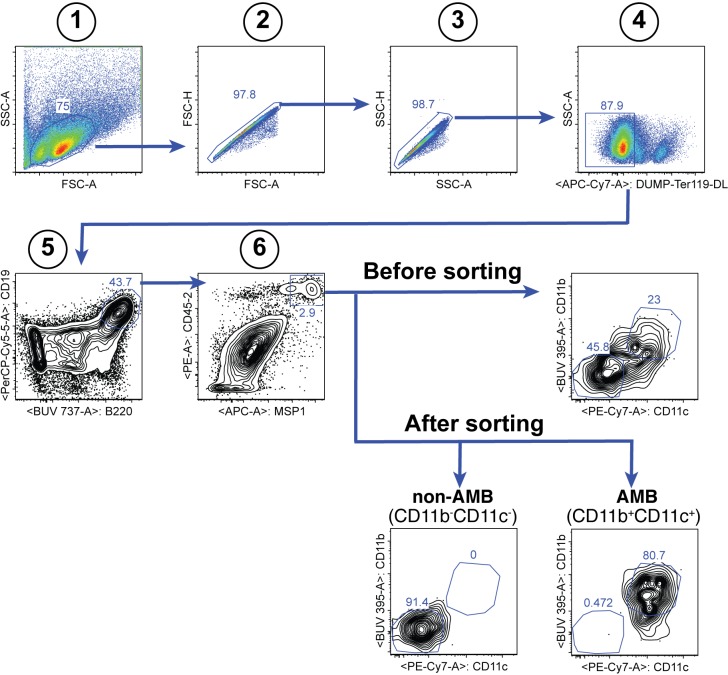
Gating strategy for the sorting of splenic MSP1_21_-specific CD11b^+^CD11c^+^ AMB and CD11b^—^CD11c^—^ B cells at 35dpi.

**Figure 3—figure supplement 2.**
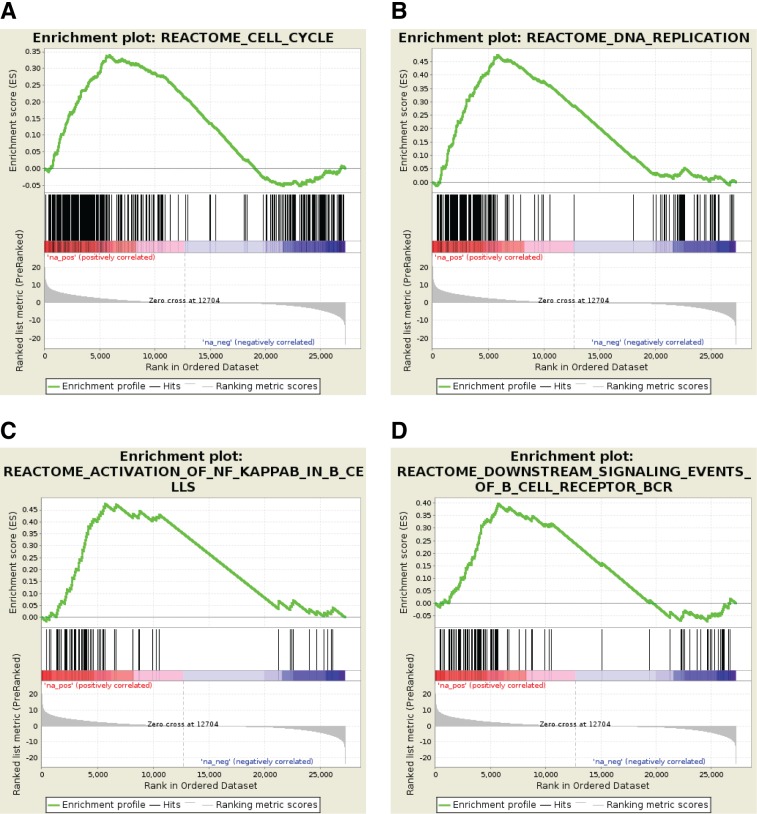
Profile of the Running *ES* Score and positions of GeneSet members on the rank ordered list for selected Reactome pathway gene sets.

We ran a Gene Set Enrichment Analysis (GSEA) (Subramanian et al., 2005) with a gene list ranked according to their differential expression between MSP1_21_-specific CD11b^+^CD11c^+^ AMB sorted from infected mice and MSP1_21_-specific B cells sorted from naïve mice, using gene sets *a priori* obtained from Reactome (Fabregat et al., 2018). Among the gene sets yielding the top 50 significant (fdr <0.001) highest normalized enrichment score (*NES*) we obtained gene sets corresponding to cell cycle, DNA replication, generation/consumption of energy, regulation of apoptosis, activation of NF-κB on B cells, and downstream signaling events of the BCR (Supplementary file 2 and Figure 3—figure supplement 2). These data further corroborate the activated and proliferative nature of MSP1_21_-specific CD11b^+^CD11c^+^ AMB.

Taken together, these data demonstrate that CD11b^+^CD11c^+^ MSP1_21_-specific mouse AMB present during the chronic phase of *P. chabaudi* infection are very similar to human AMB described in several chronic infections. In addition, this B-cell subset shows features of activation, proliferation, DNA replication and plasmablasts, resembling previous observations in human AMB (Muellenbeck et al., 2013).

### Generation of *Plasmodium*-specific AMB in response to immunization

**Figure 4.**
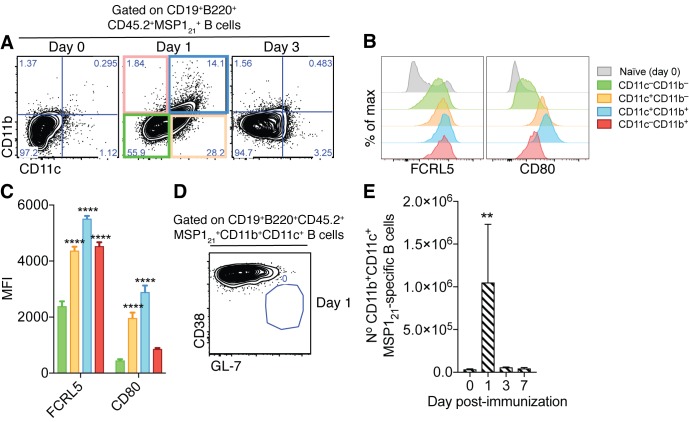
Generation of splenic MSP1_21_-specific CD11b^+^CD11c^+^AMB in response to immunization. (**A**) Flow cytometry showing differential expression of CD11b and CD11c on splenic MSP1_21_-specific B cells from *Igh*^NIMP23/+^ mice before immunization (day 0) and at days 1 and 3 post-immunization with R848 and MSP1_21_. (**B**) Flow cytometry showing expression of FCRL5 and CD80 on different subsets of splenic MSP1_21_-specific B cells from *Igh*^NIMP23/+^ defined based on CD11b and CD11c expression at day one post-immunization and naïve mice. (**C**) Geometric MFI of FCRL5 and CD80 expression on different subsets of splenic MSP1_21_-specific B cells from *Igh*^NIMP23/+^ defined based on CD11b and CD11c expression at day one post-immunization. Two-way ANOVA vs CD11b^—^CD11c^—^ subset. ****p<0.0001. (**D**) Flow cytometry of CD38 vs GL-7 (GC markers) on CD11b^+^CD11c^+^ MSP1_21_-specific B cells from *Igh*^NIMP23/+^ at day one post-immunization. (**E**) Numbers of splenic CD11b^+^CD11c^+^ MSP1_21_-specific B cells from *Igh*^NIMP23/+^ during the course of immunization. Kruskal-Wallis test compared to day 0. **p<0.01. Error bars are SEM. Data pooled from three independent experiments with 3–5 mice per group.

The occurrence of CD11b^+^CD11c^+^ AMB might be a consequence of aberrant B-cell activation driven exclusively by certain pathogens. Alternatively, they might be part of a normal B-cell response, which is exacerbated by the persistent nature of certain infections. To test whether CD11b^+^CD11c^+^FCRL5^+^ AMB could be generated in the absence of persistent infection, we immunized mice with MSP1_21_. A previous report had demonstrated the presence of CD11b^+^CD11c^+^Tbet^+^ B-cells 24 hr post-immunization with R848, a TLR7/8 ligand (Rubtsova et al., 2013). Therefore, we immunized *Igh*^NIMP23/+^ mice with R848 together with the antigen MSP1_21_ and looked for the appearance of MSP1_21_-specific CD11b^+^CD11c^+^FCRL5^+^ atypical B cells. We observed substantial numbers of MSP1_21_-specific CD11b^+^CD11c^+^ B cells in the spleens of *Igh*^NIMP23/+^ mice 24 hr post-immunization (Figure 4A). These cells expressed increased levels of both FCRL5 and CD80 (Figure 4B–C) and did not display GC characteristics (Figure 4D), similar to the MSP1_21_-specific CD11b^+^CD11c^+^FCRL5^+^ atypical B cells generated following *Plasmodium* infection. The MSP1_21_-specific CD11b^+^CD11c^+^ B cells observed after immunization appeared only transiently, as they could no longer be detected at 3 and 7d post-immunization (Figure 4A and E).

These data demonstrate that MSP1_21_-specific CD11b^+^CD11c^+^ AMB with no functional characteristics of memory B cells can be generated independently of the infection and the presence of the pathogen, and that they are short-lived cells.

### *Plasmodium*-specific CD80^+^CD273^+^ B_mem_ are generated and persist after resolution of *P. chabaudi* infection

Identification of mouse B_mem_ by flow cytometry originally relied on detecting B cells that had undergone Ig class-switching from IgM to IgG, and that did not express GC markers (i.e. IgG^+^CD38^hi^GL-7^lo^) (Lalor et al., 1992; Ridderstad and Tarlinton, 1998). More recently, this set of markers has been extended to include CD80, CD273 (PD-L2) and CD73, with CD273 and CD80 being the most useful to discriminate memory from naïve B cells (Tomayko et al., 2010; Zuccarino-Catania et al., 2014). In combination, these markers allow the identification of different subsets of switched as well as non-class switched (i.e. IgM/D^+^) B_mem_. Therefore, we used cell surface expression of CD80 and CD273 on MSP1_21_-specific B cells to identify B_mem_ during and after resolution of P. *chabaudi* infection.

**Figure 5.**
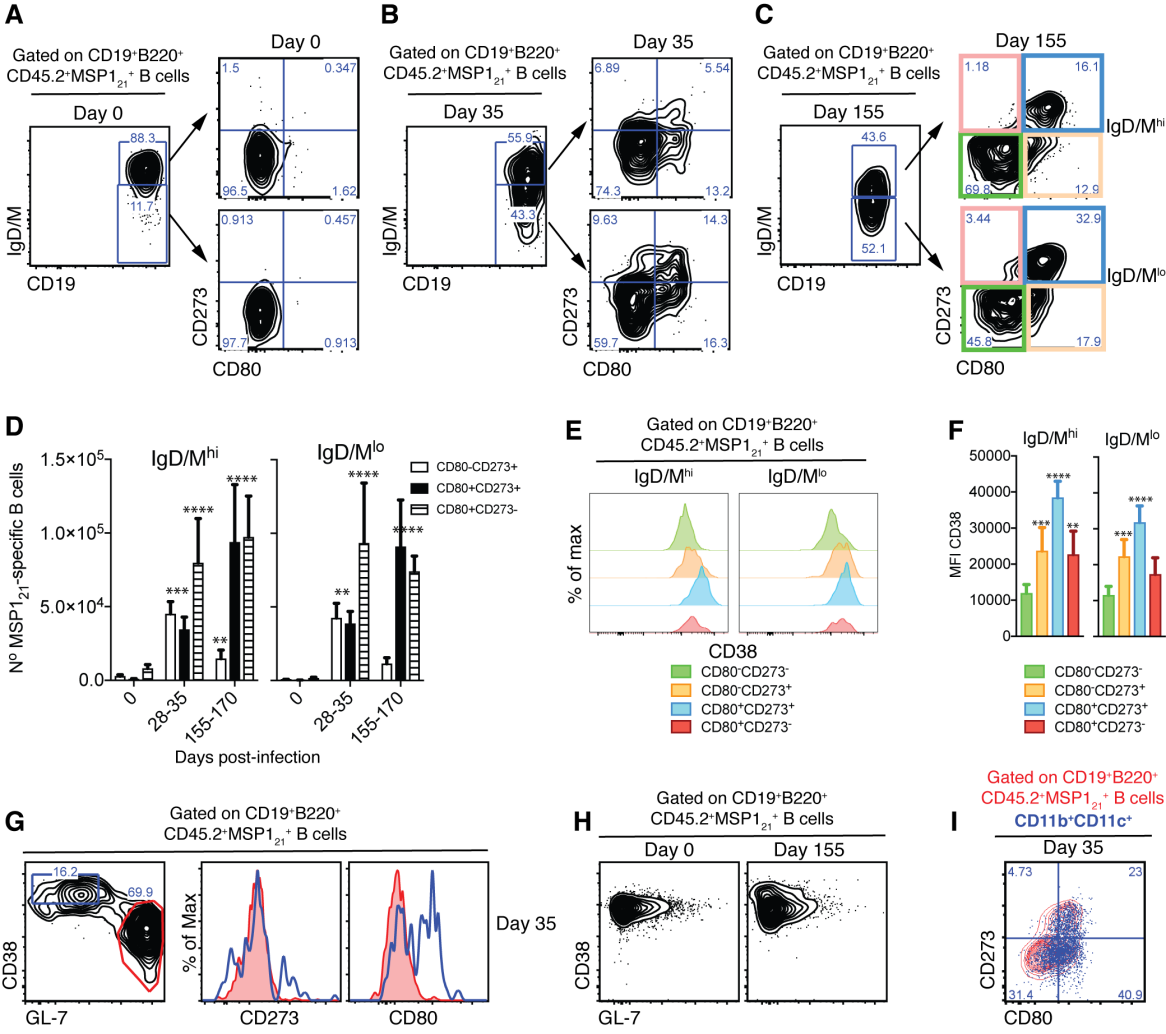
Detection of MSP1_21_-specific B_mem_ after resolution of *P.chabaudi* infection. (**A**), (**B**) and (**C**) Flow cytometry showing gating strategy to identify splenic IgM/D^hi^ and IgM/D^lo^ CD273^+^ and/or CD80^+^ MSP1_21_-specific B_mem_ in NIMP23→*Rag2^-/-^* chimeric mice before infection (day 0), at 35 and 155dpi, respectively. (**D**) Numbers of splenic IgM/D^hi^ and IgM/D^lo^ CD273^+^ and/or CD80^+^ MSP1_21_-specific B_mem_ in NIMP23→*Rag2^-/-^* chimeric mice during the course of mosquito transmitted *P. chabaudi* infection. Two-way ANOVA vs day 0. **p<0.01; ***p<0.001; ****p<0.0001. (**E**) Flow cytometry showing expression of CD38 on different subsets of splenic IgM/D^hi^ and IgM/D^lo^ MSP1_21_-specific B cells from NIMP23→*Rag2^-/-^* chimeric mice defined based on CD273 and CD80 expression at 155dpi. (**F**) Geometric MFI of CD38 expression on different subsets of IgM/D^hi^ and IgM/D^lo^ splenic MSP1_21_-specific B cells from NIMP23→*Rag2^-/-^* chimeric mice defined based on CD273 and CD80 expression at day 155 post-mosquito transmitted *P. chabaudi* infection. Two-way ANOVA vs CD273^-^CD80^-^ subset. **p<0.01; ***p<0.001; ****p<0.0001. Error bars are SEM. (**G**) Flow cytometry of CD273 and CD80 expression on non-GC (CD38^hi^GL-7^lo^, blue) and GC (CD38^lo^GL-7^hi^, red) splenic MSP1_21_-specific B cells from NIMP23→*Rag2^-/-^* chimeric mice at 35dpi. (**H**) Flow cytometry of CD38 vs GL-7 (GC markers) on splenic MSP1_21_-specific B cells from NIMP23→*Rag2^-/-^* chimeric mice at 0 and 155dpi. (**I**) CD11b^+^CD11c^+^ MSP1_21_-specific B cells overlaid on the CD80 vs CD273 plot corresponding to total MSP1_21_-specific B cells. Data pooled from three independent experiments with 3–7 mice per group.

MSP1_21_-specific B cells from spleens of naïve NIMP23→*Rag2^-/-^* mixed BM chimeras showed little expression of either CD80 or CD273 (Figure 5A). By contrast, CD80^+^CD273^+^, CD80^+^CD273^—^ and CD80^—^CD273^+^ MSP1_21_-specific B cells, both class-switched (IgM/D^lo^) and non-class-switched (IgM/D^hi^), were readily detected above background levels at 28-35dpi in *P. chabaudi*-infected NIMP23→*Rag2^-/-^* mixed BM chimeras (Figure 5B and D). After resolution of infection (155-170dpi), the numbers of CD80^+^CD273^+^ and CD80^+^CD273^—^ MSP1_21_-specific B cells were either sustained or increased, while the CD80^—^CD273^+^ population decreased, compared with 28-35dpi (Figure 5A–D). All three MSP1_21_-specific B_mem_ subsets showed high CD38 expression (Figure 5E,F). Importantly, the MSP1_21_-specific GC B cells detected at 28-35dpi did not express either CD80 or CD273, further distinguishing GC from memory and atypical memory *P. chabaudi*-specific B-cell subsets (Figure 5G). No MSP1_21_-specific GC B cells were detected above background level after resolution of the infection (Figure 5H). Finally, and in accordance with Figure 3, approximately 70% of the MSP1_21_-specific CD11b^+^CD11c^+^ AMB observed at days 28-35pi expressed either CD80, CD273 or a combination of both (Figure 5I).

These data show that, in contrast to the transient AMB, splenic CD80^+^CD273^+^ and CD80^+^CD273^—^ class-switched and non-class-switched MSP1_21_-specific B_mem_ persist after resolution of *P. chabaudi* infection.

### *Plasmodium*-specific B_mem_ express high levels of FCRL5

**Figure 6.**
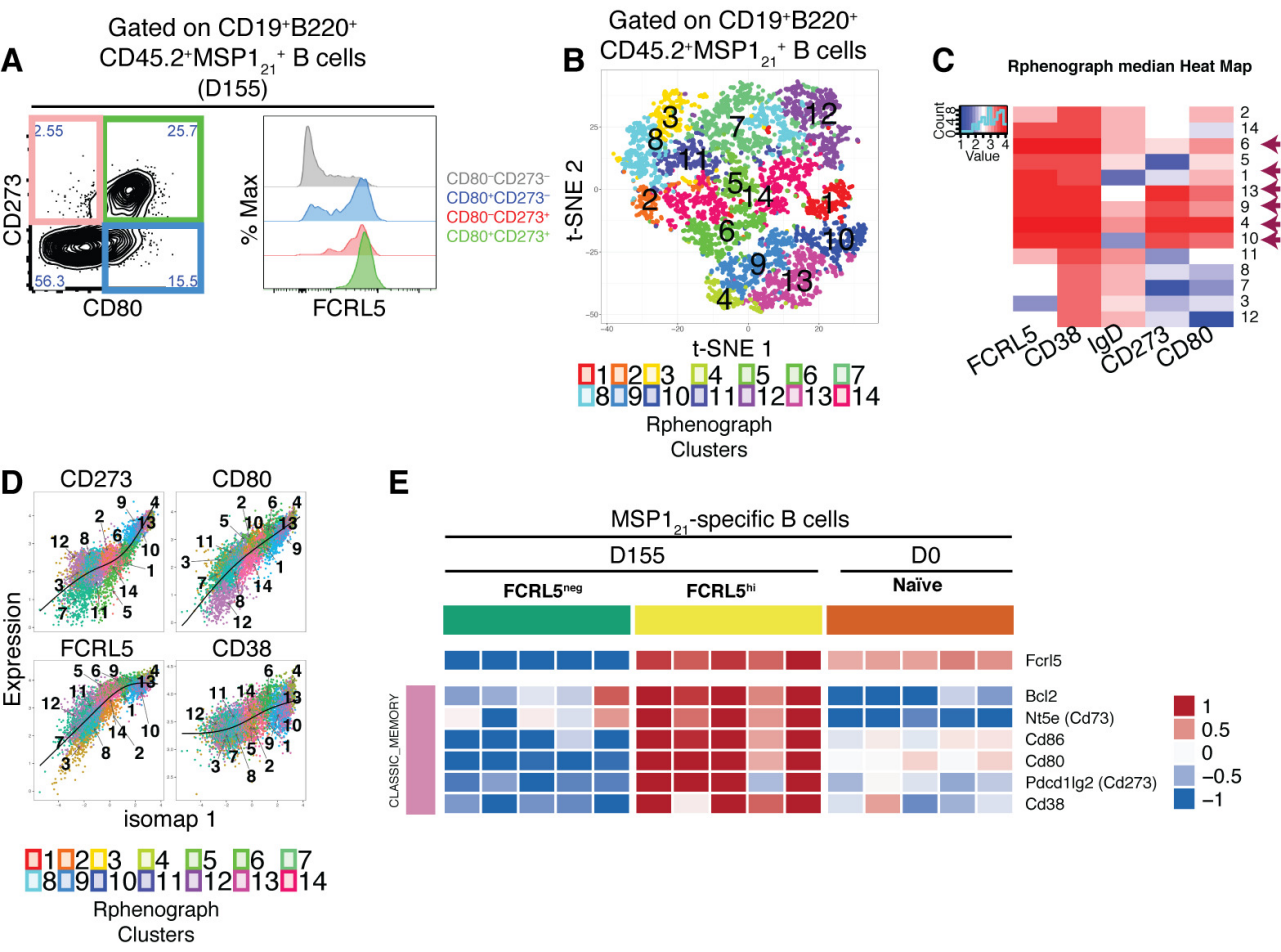
FCRL5^hi^ identifies MSP1_21_-specific B_mem_ after resolution of *P.chabaudi* infection. (**A**) Flow cytometry showing expression of FCRL5 (right) on different subsets of splenic MSP1_21_-specific B cells from NIMP23→*Rag2^-/-^* chimeric mice defined based on CD273 and CD80 expression (left) at 155dpi. (**B**) t-SNE analysis of splenic MSP1_21_-specific B cells based on FCRL5, CD38, IgD, CD273 and CD80 expression measured by flow cytometry (n = 5). Clusters identified by PhenoGraph are colored and numbered. (**C**) PhenoGraph heat map showing median expression of FCRL5, CD38, IgD, CD273 and CD80 on the different clusters of MSP1_21_-specific B cells. Arrows point at the different clusters displaying a memory B cell phenotype. (**D**) Expression profiles of FCRL5, CD38, CD273 and CD80 for the different PhenoGraph clusters visualized on the first component of ISOMAP. The regression line estimated using the generalized linear model (GLM) is added for each marker. Data representative of three independent experiments with 4–7 mice per group. (**E**) Heat map showing expression levels of different genes on splenic FCRL5^—^ and FCRL5^hi^ MSP1_21_-specific B cells sorted at 155dpi, and MSP1_21_-specific B cells sorted before infection (naïve), determined by RNAseq analysis. Each column corresponds to data from an individual mouse (n = 5 155 dpi, n = 5 0 dpi).

As discussed above, no single marker has been described so far that can identify all mouse B_mem_ subsets. Surprisingly, we observed that after resolution of infection (155-170dpi), MSP1_21_-specific B cells expressing different combinations of CD80 and CD273 (CD80^+^CD273^+^, CD80^-^CD273^+^ or CD80^+^CD273^-^) all expressed very high levels of FCRL5, in contrast to CD80^-^CD273^-^ MSP1_21_-specific B cells that express no memory markers at this stage (Figure 6A). This suggests that FCRL5 might be a marker for all B_mem_. In order to confirm this, we used unsupervised methods to analyze our multiparameter flow cytometry data. We used PhenoGraph and t-SNE within the Cytofkit package (Materials and methods, Chen et al 2016) to analyze MSP1_21_-specific B cells based on the expression of FCRL5, CD38, IgD, CD80 and CD273 on these cells, as determined by flow cytometry (Figure 6A and B). The analysis identified six clusters of cells with memory characteristics displaying high expression of CD38, CD80 and/or CD273, and variable expression of IgD, all of which expressed high levels of FCRL5 (Figure 6C: clusters identified with purple arrows). We then used Isomap, (Cytofkit package) to infer the relatedness between those cell subsets identified by PhenoGraph. This confirmed high similarities between the cell clusters expressing high levels of FCRL5 with the clusters expressing high levels of the memory markers CD80, CD273 and CD38 (Figure 6D).

**Figure 6—figure supplement 1.**
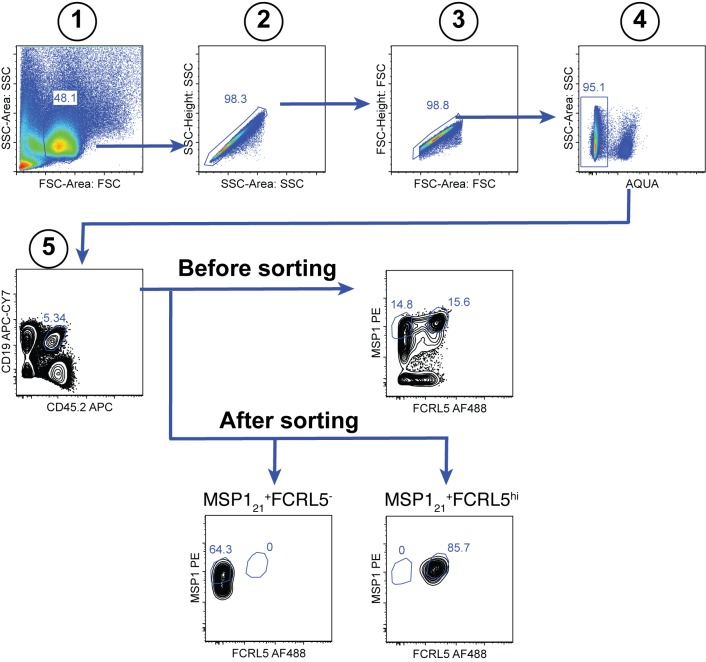
Gating strategy for the sorting of splenic MSP1_21_-specific FCRL5^hi^ B_mem_ and FCRL5^—^ B cells at 155dpi.

**Figure 6—figure supplement 2.**
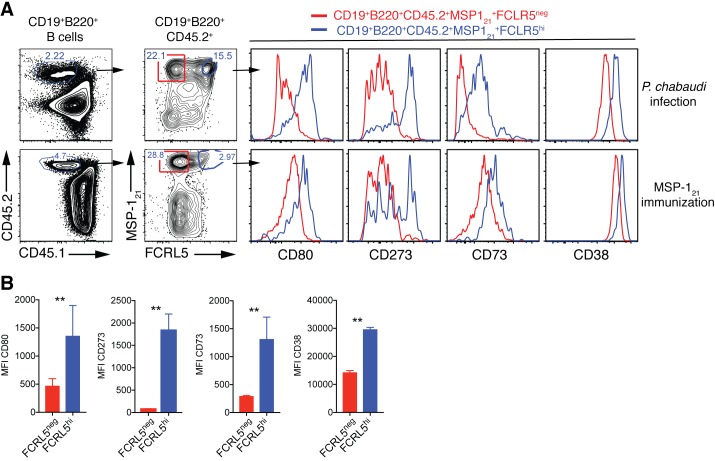
High expression of FCRL5 identifies B_mem_. (**A**) Flow cytometry gating strategy to identify splenic MSP1_21_-specific FCRL5^neg^ (red) and FCRL5^hi^ (blue) B cells, and additional surface markers expressed on these cells, in NIMP23→ *Rag2^-/-^* mixed bone marrow chimeras infected with *P. chabaudi* (155dpi, top row) and immunized with recombinant MSP1_21_ in Titermax Gold emulsion (day 29 post-immunization, bottom row). (**B**) Cumulative data showing geometric MFI for CD80, CD273, CD73, and CD38 memory markers on splenic MSP1_21_-specific FCRL5^neg^ and FCRL5^hi^ B cells obtained from immunized mice. Mann Whitney U test. **, p<0.01. Error bars are SEM. Data representative of two independent experiments with 3–5 mice per group.

To confirm the memory identity of MSP1_21_-specific FCRL5^hi^ B-cells detected after resolution of the infection, we isolated MSP1_21_-specific B cells expressing either high levels of FCRL5 or not expressing FCRL5 (i.e. FCRL5^hi^ and FCRL5^—^ MSP1_21_-specific B cells) from the spleen of *P. chabaudi*-infected NIMP23→*Rag2^-/-^* mice (155dpi) (Figure 6—figure supplement 1), and MSP1_21_-specific B cells from the spleen of naïve NIMP23→*Rag2^-/-^* mice, by flow cytometric sorting, and performed mRNAseq analysis on these three sorted cell populations (Figure 6E). As expected, the MSP1_21_-specific FCRL5^hi^ B cell subset showed high expression of genes encoding the hallmark memory B cell markers *Cd38*, *Cd80*, *Cd86*, *Nt5e* (CD73) and *Pdcd1lg2* (CD273), when compared with either MSP1_21_-specific FCRL5^—^ B cells sorted at the same time or MSP1_21_-specific B cells sorted from naïve mice (Figure 6E). Moreover, the MSP1_21_-specific FCRL5^hi^ B cells sorted after resolution of the infection upregulated the anti-apoptotic *Bcl2* gene, which is an additional hallmark characteristic of memory B cells (Figure 6E). Importantly, FCRL5 also identified CD80^+^ and CD273^+^ MSP1_21_-specific B_mem_ subsets generated following immunization with a model antigen (Figure 6—figure supplement 2).

Thus, after resolution of the infection, high expression of FCRL5 identifies *P. chabaudi*-specific B_mem_.

### *Plasmodium*-specific AMB are a distinct short-lived activated B cell subset

**Figure 7.**
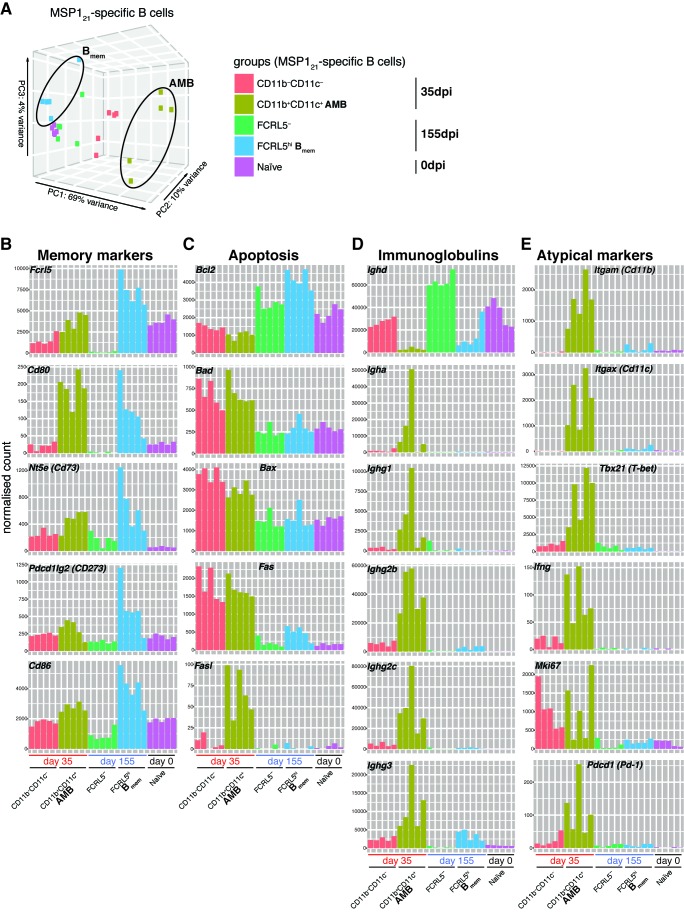
MSP1_21_-specific AMB are a distinct short-lived activated B cell subset. (**A**) Principal component analysis of RNAseq transcriptome data from splenic MSP1_21_-specific AMB (CD11b^+^CD11c^+^, 35dpi), CD11b^—^CD11c^—^ B cells (35dpi), B_mem_ (FCRL5^hi^, 155dpi), FCRL5^—^ B cells (155dpi) and B cells from naïve mice (0dpi). The MSP1_21_-specific AMB and B_mem_ are contained inside ellipses. (**B**) (**C**) (**D**) (**E**) Normalized counts corresponding to selected genes representing memory B-cell markers, anti and pro-apoptotic genes, immunoglobulins and atypical memory B-cell markers, respectively, for all five groups described in (**A**). Each bar represents an individual mouse. Data generated with five mice per group.

After identifying and sorting MSP1_21_-specific AMB during chronic *P. chabaudi* infection, and MSP1_21_-specific B_mem_ after resolution of the infection, we then compared the transcriptome of these two B-cell subsets. Principal component analysis (PCA) demonstrated a strikingly distinct transcriptome of MSP1_21_-specific AMB from that of MSP1_21_-specific B_mem_, as well as all other MSP1_21_-specific B-cell subsets sorted in this study (Figure 7A). The MSP1_21_-specific AMB sorted at 35dpi formed a separated cluster at the extreme right of the PC1 axis of the PCA plot, which accounts for the majority of the variance (Figure 7A). All the other subsets [including MSP1_21_-specific CD11b^—^CD11c^—^ B-cells sorted from the same mice and at the same day post-infection as the MSP1_21_-specific AMB (i.e. 35dpi)] clustered on the left of the PC1 axis, and showed differences mostly along the PC2 axis of the PCA plot, which accounts for only 10% of the variance (Figure 7A). Interestingly, MSP1_21_-specific CD11b^—^CD11c^—^ and MSP1_21_-specific B_mem_ clustered on opposite sides of the MSP1_21_-specific naïve B-cell subset along the PC2 axis (Figure 7A), which suggests that the MSP1_21_-specific B_mem_ more closely resemble MSP1_21_-specific naïve B cells than MSP1_21_-specific CD11b^—^CD11c^—^ B cells sorted at 35dpi.

MSP1_21_-specific AMB sorted during chronic *P. chabaudi* infection, and MSP1_21_-specific B_mem_ sorted after resolution of the infection shared the expression of a series of mouse memory markers, including *Cd80*, *Fcrl5*, *Nt5e* (CD73), and *Cd86* (Figure 7B). However, these two subsets showed differences in the expression pattern of anti- and pro-apoptotic genes (Figure 7C). While MSP1_21_-specific B_mem_ from after infection resolution showed the highest levels of expression of the anti-apoptotic *Bcl2* gene, MSP1_21_-specific AMB sorted during chronic *P. chabaudi* infection showed the lowest levels of expression of this hallmark anti-apoptotic gene (Figure 7C). In contrast to MSP1_21_-specific B_mem_, MSP1_21_-specific AMB expressed high levels of the pro-apoptotic genes *Bad*, *Bax*, *Fas* and *Fasl* (Figure 7C). Interestingly, MSP1_21_-specific AMB expressed very high levels of class-switched immunoglobulins, including *Igha*, *Ighg1*, *Ighg2b*, *Ighg2c* and *Ighg3* (Figure 7D). Finally, MSP1_21_-specific AMB highly expressed *Cd11b*, *Cd11c*, *Tbx21*, *Ifng* and *Pdcd1* (Figure 7E), all hallmarks of human AMB, as well as *Mki67*, indicative of active cell division, as previously shown in human AMB.

These data demonstrate that AMB and B_mem_ share the expression of memory markers. However, they show striking differences in the expression of pro- and anti-apoptotic genes, immunoglobulins genes, and cell proliferation genes. The increased expression of *Mki67*, pro-apoptotic genes and class-switched immunoglobulins in AMB suggests that they resemble activated B cells. By contrast, B_mem_ express much less *Mki67* (similar to naïve cells) and present an anti-apoptotic gene expression pattern, consistent with being long-lived quiescent B cells.

**Figure 8.**
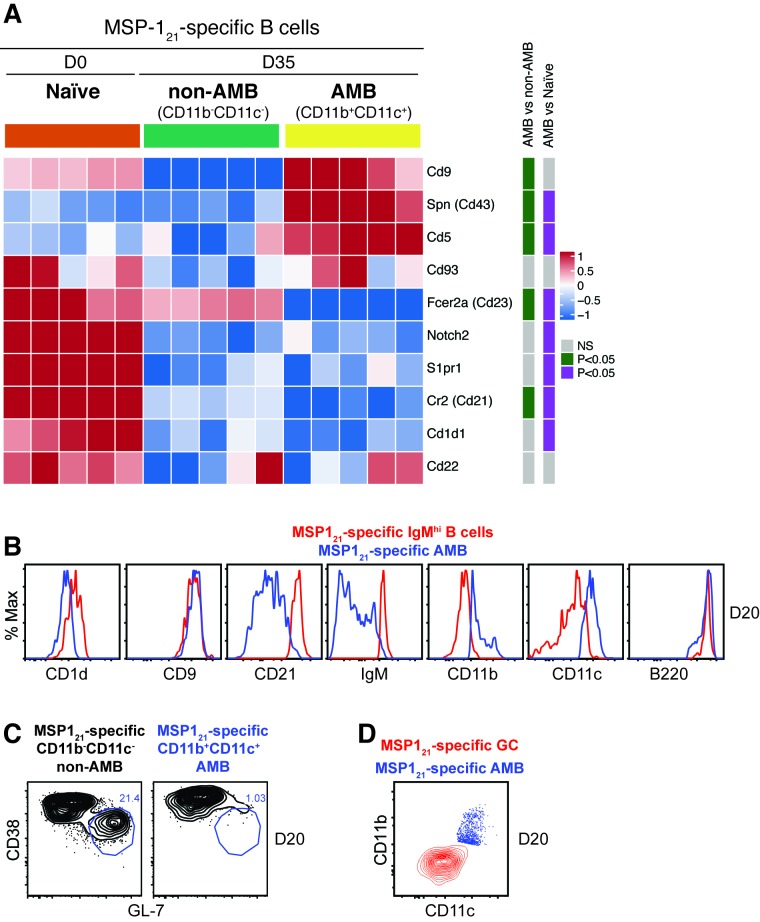
Analysis of MZ, B1 and GC B cell characteristics on MSP1_21_-specific AMB. (**A**) MSP1_21_-specific CD11b^+^CD11c^+^ (AMB) and CD11b^—^CD11c^—^ B cells were flow cytometry sorted from the spleen of NIMP23→*Rag2^-/-^* chimeric mice at 35dpi; MSP1_21_-specific B cells were flow cytometry sorted from the spleen of naïve NIMP23→*Rag2^-/-^*, and these three B cell populations were submitted to mRNAseq analysis. The heat map displays level of expression of selected individual genes known to be up or downregulated on either MZ, B1 B cells or both. Each column represents an individual mouse. (**B**) Flow cytometry analysis of surface markers of either MZ, B1 B cells or both, on MSP1_21_-specific CD11b^+^CD11c^+^ (AMB) (blue) and MSP1_21_-specific IgM^hi^ (red) B cells from the spleen of *Igh*^NIMP23/+^ mice at 20dpi. (**C**) Flow cytometry analysis of GC markers on MSP1_21_-specific CD11b^—^CD11c^—^ (non-AMB, left) and CD11b^+^CD11c^+^ (AMB, right) B cells from the spleen of *Igh*^NIMP23/+^ mice at 20dpi. (**D**) Flow cytometry analysis showing the expression of CD11b and CD11c on MSP1_21_-specific CD11b^+^CD11c^+^ (AMB, blue) compared to GC (CD38^lo^GL-7^hi^, red) B cells from the spleen of *Igh*^NIMP23/+^ mice. Data generated with 5–6 mice per group.

Mouse FCRL5 has been shown to be expressed on marginal zone (MZ) and B1 B cells (Won et al., 2006). Therefore, it is possible that the AMB identified in this study might represent a specific subset of either MZ or B1 B cells. As discussed elsewhere (Baumgarth, 2011; Garraud et al., 2012; Pillai et al., 2005; Zouali, 2011), B1 and MZ B cells are CD1d^mid/hi^, CD9^+^, IgM^hi^ and CD23^—^. B1 are further CD43^+^, B220^lo^, and may (B1a) or may not (B1b) express CD5. MZ are further characterised by CD22^hi^, CD21/CR2^hi^, and the expression of the lysophospholipid sphingosine-1 phosphate receptor S1P_1_, and the lineage master regulator Notch2. Indeed, MSP1_21_-specific CD11b^+^CD11c^+^ AMB showed high expression of some markers associated with B1 and/or MZ, including CD5, CD9 and CD43. However, these markers have also been shown to be highly expressed by activated B2 B cells and plasma cells (Baumgarth, 2011). Studying all these canonical markers, we found no other similarities between B1/MZ and MSP1_21_-specific CD11b^+^CD11c^+^ AMB to suggest a relationship between these subsets (Figure 8A). This was further corroborated by flow cytometry analysis (Figure 8B). Moreover, unlike MZ and B1 B cells, MSP1_21_-specific AMB were class-switched and expressed very high levels of IgG (Figure 7D). To further characterize the identity of MSP1_21_-specific CD11b^+^CD11c^+^ AMB, we explored if these cells resemble GC B cells. Flow cytometry analysis demonstrated that MSP1_21_-specific AMB did not display a GC phenotype (i.e. CD38^hi^GL-7^lo^) (Figure 8C) while the MSP1_21_-specific CD38^lo^GL-7^hi^ GC B cells showed low expression of both CD11b and CD11c. Thus, MSP1_21_-specific AMB are not GC B cells.

All together, these data further show that *P. chabaudi*-specific AMB represent a distinct subset of short-lived activated B cells.

## Discussion

Similar to other chronic infections [e.g. HIV, HCV and *Mycobacterium tuberculosis* (Knox et al., 2017b; Portugal et al., 2017)], *Plasmodium* infection, the cause of malaria, leads to an increase in the frequency of AMB [originally termed tissue-like memory B cells (Moir et al., 2008)] in peripheral blood from *P. falciparum*-exposed subjects (Illingworth et al., 2013; Portugal et al., 2015; Sullivan et al., 2016; Sullivan et al., 2015; Weiss et al., 2011; Weiss et al., 2010; Weiss et al., 2009). However, mouse models to investigate *Plasmodium*-specific AMB are lacking. Here we have generated an IgH knock-in transgenic mouse strain to study the generation of *Plasmodium*-specific AMB in a *Plasmodium chabaudi* infection. We demonstrate the generation of *P. chabaudi*-specific AMB in response to blood-stage mosquito-transmitted chronic *P. chabaudi* infection, and show the short-lived nature of these cells. *P. chabaudi* infections in mice present some outstanding characteristics for the study of AMB; a chronic phase which allows investigation of the impact of constant immune activation driven by persistent subpatent parasitemia, followed by a clearance phase which allows the study of immune responses after the infection is naturally resolved. Thus, it is possible to study both exhaustion driven by chronic immune activation, and memory immune responses which remain after *P. chabaudi* elimination.

The *P. chabaudi*-specific AMB we detected during the chronic phase of infection showed strong similarities to human AMB described in chronic malaria. These included being class-switched, and expressing mouse homologues of hallmark human atypical memory B-cell genes such as *Itgax* (Cd11b), *Itgam* (Cd11c), *Cxcr3*, *Fcrl5*, *Tbx21* (T-bet), *Ifng*, *Cd80*, *Cd86*, *Aicda*, and a series on inhibitory receptors, including *Lair1* and *Pdcd1* (PD-1). Human AMB have been shown to share several characteristics with plasmablasts (Knox et al., 2017a; Muellenbeck et al., 2013; Obeng-Adjei et al., 2017; Sullivan et al., 2015). Interestingly, we also observed increased expression of several genes associated with plasmablasts in *P. chabaudi*-specific AMB, including *Cd138*, *Xbp1*, *Prdm1* (encoding Blimp-1) and *Mki67*, accompanied by downregulation of *Pax5* and *Bcl6*.

AMB resemble age-associated B cells (ABC) which accumulate with age as well as in autoimmunity, and were also proposed to be a subset of long-lived memory B cells (Naradikian et al., 2016a; Portugal et al., 2017; Rubtsov et al., 2017). Expression of T-bet, CD11c and CXCR3 are shared by AMB, tissue-like memory B cells, and ABC (Knox et al., 2017b; Naradikian et al., 2016a). Moreover, similar to ABC, expansion of human AMB associated with malaria is driven by IFNγ (Obeng-Adjei et al., 2017). IL-21, which is highly expressed by follicular helper T cells in response to *Plasmodium* infection (Carpio et al., 2015; Obeng-Adjei et al., 2015; Pérez-Mazliah et al., 2015), also directly promotes T-bet expression in B cells in the context of TLR engagement (Naradikian et al., 2016b). Taken together, these data strongly suggest that this is the same T-bet^+^ B-cell subset, which accumulates with time due to repetitive antigenic exposure. In agreement with previous data (Rubtsova et al., 2013), we show here that immunization with MSP1_21_ and R848, a TLR7/8 ligand, promotes a robust but short-lived CD11b^+^CD11c^+^ *P. chabaudi*-specific AMB response. T-bet^+^ atypical B cells are critical to eradicate various murine viral infections (Barnett et al., 2016; Rubtsova et al., 2013), and a recent study showed that yellow fever and vaccinia vaccinations of humans stimulated an acute T-bet^+^ B-cell response and suggested that these T-bet^+^ B-cell population may function as an early responder during acute viral infections (Knox et al., 2017a). Thus, T-bet^+^ B cells, even in the context of malaria, are likely to be a normal component of the immune compartment that becomes activated and expands, most probably in response to BCR, endosomal TLR, and IFNγ or IL-21 stimulation. Moreover, a recent study shows a TLR9/IFNγ-dependent activation of autoreactive T-bet^+^CD11c^+^ atypical B cells in response to *P. yoelii* 17XNL infection in mice (Rivera-Correa et al., 2017). However, whether these cells remained part of the long-lived memory B-cell pool after resolution of the infection was not explored.

Here, we show that *P. chabaudi*-specific AMB are short-lived activated B cells. These cells were absent after resolution of the infection, and immunization with purified antigen and TLR agonists resulted in a transient, yet robust, activation of *P. chabaudi*-specific AMB which lasted no more than 48 hr. Moreover, the *Igh*^NIMP23/+^ mouse model allowed us to obtain a deep insight of the transcriptome profile of MSP1_21_-specific AMB during natural infection, and compare it side-by-side with the transcriptome of MSP1_21_-specific naïve B cells. This allowed us to demonstrate their heavily pro-apoptotic and activated transcription profile, further explaining their short-lived nature. *P. chabaudi*-specific AMB showed very low expression of *Bcl2*, and high levels of expression of several pro-apoptotic genes including *Bad*, *Bax*, *Fas* and *Fasl.* In addition, these cells expressed very high levels of class-switched immunoglobulins and genes associated with DNA replication and proliferation.

The association of *P. chabaudi*-specific AMB with ongoing infection explains several observations in human studies: reduction of HIV plasma viremia by ART resulted in a significant reduction of HIV-specific AMB without altering the frequency of HIV-specific B_mem_ (de Bree et al., 2017; Kardava et al., 2014); individuals living in high malaria endemicity present higher frequencies of AMB than individuals living in areas with moderate transmission (Illingworth et al., 2013; Sullivan et al., 2015); repetitive *Plasmodium* episodes result in higher frequencies of AMB (Obeng-Adjei et al., 2017); the percentage of AMB is larger in children with persistent asymptomatic *Plasmodium falciparum* parasitemia as compared with parasite-free children (Weiss et al., 2009); previously exposed subjects significantly reduce the frequency of AMB following a year of continuous absence of exposure to *Plasmodium falciparum* infection (Ayieko et al., 2013). These observations all support the view that constant immune activation rather than impaired memory function leads to the accumulation of AMB in malaria.

After resolution of infection, *P. chabaudi*-specific AMB did not persist, but instead subsets of *P. chabaudi*-specific B_mem_ were readily detected. These cells expressed different combinations of previously described mouse B-cell memory markers [i.e. CD80, CD273 and CD73 (Anderson et al., 2007; Tomayko et al., 2010; Zuccarino-Catania et al., 2014)]. *P. chabaudi*-specific B_mem_ included both class-switched and non-class-switched cells, which show different responses to a secondary challenge infection (Krishnamurty et al., 2016). Independently of the combination of previously described memory markers expressed on *P. chabaudi*-specific B_mem_, all of these cells displayed very high expression of FCRL5. Previous data showed most prominent expression of FCRL5 in marginal zone B cells, while much less evident in the newly-formed and follicular splenic B cell subpopulations (Davis et al., 2004; Won et al., 2006). In agreement with this, we observed expression of FCRL5 on a subset of splenic *P. chabaudi*-specific B cells obtained from naïve mice. However, the level of FCRL5 expression on *P. chabaudi*-specific B_mem_ detected after resolution of the infection was noticeably higher than that of naïve B cells both at the protein and mRNA levels. In contrast to MSP1_21_-specific AMB, these CD11b^—^CD11c^—^FCRL5^hi^ MSP1_21_-specific B_mem_ showed high expression of the hallmark memory and anti-apoptotic gene *Bcl2* (Bhattacharya et al., 2007). Thus, after resolution of the infection, high expression of FCRL5 acted as a universal B_mem_ marker. Due to its complex dual ITIM/ITAM signaling capacity (Zhu et al., 2013), it is tempting to speculate that FCRL5 might serve as an important signal in the differentiation/maintenance of B_mem_.

Tracking the fate of the different MSP1_21_-specific B-cell subsets identified in this work will allow detailing the interplay between them. A model can be proposed in which antigen-specific AMB serve as an intermediate stage of differentiation between naïve and B_mem_. Alternatively, antigen-specific AMB might represent early plasmablasts or recent GC emigrates, based on class-switching and the high expression of IgG by these cells. These scenarios are not necessarily antagonist, and might even occur in parallel. Moreover, B1 B cells have been shown to class-switch and contribute to serum IgG1, IgG2a and IgA to influenza (Baumgarth et al., 2005), and IgG-producing B1a B cells have been shown to accumulate in the spleen of a mouse model of systemic lupus erythematosus (Enghard et al., 2010). Therefore, we can’t rule out the possibility that AMB might represent a particular B1 B cell subset that expands in the spleen and blood in response to *Plasmodium* infection.

Our data suggest that the expansion of AMB in malaria is not a consequence of B-cell exhaustion, but rather a physiologic stage of B-cell activation, and that these cells are sustained in high frequencies by ongoing chronic infections. Thus, *Plasmodium*-specific AMB are neither ‘memory’, nor ‘atypical’. Importantly, our data demonstrate that robust expansion of *Plasmodium*-specific AMB does not hinder clearance of the infection, activation of germinal centers, or generation of *Plasmodium*-specific long-lived quiescent B_mem_ upon resolution of the infection.

## Materials and methods

**Key resources table:** 

Reagent type (species) or resource	Designation	Source or reference	Identifiers	Additional information
Genetic reagent (*M. musculus*)	B6.SJL-Ptprc^a^ Pepc^b^/BoyJ (B6.CD45.1)	The Jackson Laboratory	MGI:4819849	Bred in the specific pathogen-free facilities of the MRC National Institute for Medical Research and The Francis Crick Institute
Genetic reagent (*M. musculus*)	Rag2^tm1Fwa^(*Rag2*^-/-^)	The Jackson Laboratory	MGI:1858556	Bred in the specific pathogen- free facilities of the MRC National Institute for Medical Research and The Francis Crick Institute
Genetic reagent (*M. musculus*)	*Igh*^NIMP23/+^	This paper	_	Bred in the specific pathogen-free facilities of the MRC National Institute for Medical Research and The Francis Crick Institute
Strain, strain background (*Plasmodium* *chabaudi* *chabaudi*, strain AS)	*P. chabaudi*	other	_	European Malaria Reagent Repository, University of Edinburgh.
Strain, strain background (*Anopheles* *stephensi*, strain SD500, female)	mosquitos	PMID: 23217144	_	Bred in Jean Langhorne's lab
Antibody	Monoclonal Rat Anti-CD11b	BD Biosciences	563553	(dil 1/50)
Antibody	Monoclonal Hamster Anti-Mouse CD11c	BD Biosciences	561022	(dil 1/50)
Antibody	Monoclonal Rat Anti-Mouse CD138	BD Biosciences	553714	(dil 1/400)
Antibody	Monoclonal Rat Anti-Mouse CD19	BD Biosciences	565076	(dil 1/200)
Antibody	Monoclonal Rat anti-Mouse CD19	Biolegend	115530	(dil 1/400)
Antibody	Monoclonal Rat anti-Mouse CD19	Biolegend	115543	(dil 1/400)
Antibody	Monoclonal Rat anti-Mouse CD1d	Biolegend	123510	(dil 1/100)
Antibody	Monoclonal Rat anti-mouse CD2	Biolegend	100112	(dil 1/100)
Antibody	Monoclona Rat Anti-Mouse CD21/35	BD Biosciences	563176	(dil 1/100)
Antibody	Monoclona Rat Anti-Mouse CD21/35	BD Biosciences	553818	(dil 1/100)
Antibody	Monoclonal Rat anti-Mouse CD23	eBioscience	25–0232	(dil 1/100)
Antibody	Monoclonal Rat anti-Mouse CD273	BD Biosciences	564245	(dil 1/25)
Antibody	Armenian Hamster anti-Mouse CD3	Biolegend	100336	(dil 1/100)
Antibody	Monoclonal Rat anti-Mouse CD38	Biolegend	102718	(dil 1/400)
Antibody	Monoclonal Rat anti-Mouse CD38	eBioscience	17–0381	(dil 1/400)
Antibody	Monoclonal Rat anti-Mouse CD38	BD Biosciences	740697	(dil 1/400)
Antibody	Monoclonal Rat anti-Mouse CD4	Biolegend	100414	(dil 1/400)
Antibody	Monoclonal Rat anti-Mouse CD45.1	Biolegend	110706	(dil 1/400)
Antibody	Monoclonal Rat anti-Mouse CD45.1	Biolegend	110728	(dil 1/400)
Antibody	Monoclonal Mouse anti-Mouse CD45.2	BD Biosciences	563685	(dil 1/50)
Antibody	Monoclonal Mouse anti-Mouse CD45.2	Biolegend	109814	(dil 1/50)
Antibody	Monoclonal Mouse anti-Mouse CD45.2	Biolegend	109808	(dil 1/50)
Antibody	Monoclonal Rat anti-Mouse CD45R/B220	BD Biosciences	564449	(dil 1/400)
Antibody	Monoclonal Rat anti-Mouse CD45R/B220	Biolegend	103224	(dil 1/400)
Antibody	Monoclonal Rat anti-Mouse CD45R/B220	eBioscience	25–0452	(dil 1/400)
Antibody	Monoclonal Rat anti-Mouse CD73	BD Biosciences	550741	(dil 1/100)
Antibody	Monoclonal Armenian Hamster anti-Mouse CD80	Biolegend	104729	(dil 1/25)
Antibody	Monoclonal Rat anti-Mouse CD8a	Biolegend	100734	(dil 1/400)
Antibody	Monoclonal Rat anti-Mouse CD9	BD Biosciences	558749	(dil 1/100)
Antibody	Monoclonal Rat anti-Mouse CD93 (AA4.1)	eBioscience	17–5892	(dil 1/100)
Antibody	FCRL5	PMID: 17082595	_	Produced in Randall Davis' lab (dil 1/400)
Antibody	Polyclonal Sheep anti-Mouse FCRL5	R and D Systems	FAB6756G	(dil 1/50)
Antibody	Monoclonal Rat Anti-Mouse T- and B-Cell Activation Antigen GL7	BD Biosciences	562080	(dil 1/100)
Antibody	Monoclonal Rat Anti-Mouse IgD	Biolegend	405725	(dil 1/400)
Antibody	Monoclonal Rat Anti-Mouse IgD	Biolegend	405723	(dil 1/400)
Antibody	Monoclonal Rat Anti-Mouse IgD	Biolegend	405710	(dil 1/400)
Antibody	Monoclonal Rat Anti-Mouse IgG2b	Biolegend	406708	(dil 1/25)
Antibody	Monoclonal Rat Anti-Mouse IgM	Biolegend	406512	(dil 1/100)
Recombinant DNA reagent	VDJ_H_^NIMP23^ anti-MSP1_21_ variable region coding exon containing the Leader-V segment intron from gDNA of the NIMP23 hybridoma	PMID: 7141700	_	Produced in Jean Langhorne's lab
Recombinant DNA reagent	C57Bl/6 IgH HEL variable region knock-in construct	PMID: 12668643	_	Donated by Robert Brink of the Garvan Institute of Medical Research, New South Wales, Australia
Peptide, recombinant protein	MSP1_21_	PMID: 11254580	_	Produced in Jean Langhorne's lab
Commercial assay or kit	EZ-Link Sulfo-NHS-LC-Biotinylation Kit	Thermo Scientific	21435	
Commercial assay or kit	RiboPure RNA Purification Kit	Invitrogen	AM1924	
Commercial assay or kit	Qubit 1X dsDNA HS Assay Kit	Invitrogen	Q33231	
Commercial assay or kit	SMART-Seq v4 Ultra Low Input RNA Kit for Sequencing	Takara	634889	
Commercial assay or kit	Ovation Ultralow Library System V2	Nugen	0344–32	
Commercial assay or kit	LIVE/DEAD Fixable Aqua Dead Cell Stain Kit	Invitrogen	L34957	
Commercial assay or kit	LIVE/DEAD Fixable Blue Dead Cell Stain Kit	Invitrogen	L23105	
Chemical compound, drug	Streptavidin-R-Phycoerythrin	Prozyme	PJRS25	
Chemical compound, drug	Streptavidin-Allophycocyanin	Prozyme	PJ27S	
Chemical compound, drug	TiterMax Gold Adjuvant	Merck (formerly Sigma-Aldrich)	T2684-1ML	
Chemical compound, drug	R848 (Resiquimod)	Invivogen	tlrl-r848	
Chemical compound, drug	TRI Reagent Solution	Invitrogen	AM9738	
Software, algorithm	cutadapt v1.9.1	doi:10.14806/ej.17.1.200		
Software, algorithm	RSEM v1.2.31	doi:10.1186/1471-2105-12-323		
Software, algorithm	STAR v2.5.1b	doi:10.1093/ bioinformatics/bts635		
Software, algorithm	DESeq2	doi:10.1186/s13059-014-0550-8		
Software, algorithm	R v3.4.0	other		https://www.r-project.org
Software, algorithm	Bioconductor v3.5	other		http://www. bioconductor.org
Software, algorithm	Broad's Gene Set Enrichment Analysis (GSEA)	other		http://software.broadinstitute.org/gsea/index.jsp
Software, algorithm	FlowJo version 9.6 or higher	Tree Star		
Software, algorithm	Cytofkit	doi:10.1371/journa l.pcbi.1005112.s009		
Software, algorithm	Prism v6	GraphPad		

### Mice

5-12 week-old female mice were used for experiments. C57BL/6J, C57BL/6.SJL-*Ptprc^a^* (CD45.1 congenic), *Rag2^-/-^*.C57BL/6.SJL-*Ptprc^a^* (CD45.1 congenic) and BALB/c mouse strains were bred in the specific pathogen-free facilities of the MRC National Institute for Medical Research and The Francis Crick Institute, and were housed conventionally with sterile bedding, food and irradiated water. Room temperature was 22°C with a 12 hr light/dark cycle; food and water were provided ad libitum. The study was carried out in accordance with the UK Animals (Scientific Procedures) Act 1986 (Home Office license 80/2538 and 70/8326), was approved by the MRC National Institute for Medical Research Ethical Committee and was approved by The Francis Crick Institute Ethical Committee.

To produce MSP1_21_–specific B cell knock-in mice capable of undergoing class switch recombination on the C57BL/6J genetic background, the VDJ_H_^NIMP23^ anti-MSP1_21_ variable region coding exon containing the Leader-V segment intron from gDNA of the NIMP23 hybridoma (Boyle et al., 1982) was inserted by homologous recombination into the 5’ end of the endogenous IgH locus (Taki et al., 1993) (Figure 1—figure supplement 1A–C). The VDJ_H_^NIMP23^ anti-MSP1_21_ variable region coding exon was inserted into a previously described IgH targeting construct, replacing the anti-HEL heavy chain variable region coding exon that was already in it (Phan et al., 2003) (Figure 1—figure supplement 1D). The final targeting construct included a loxP-flanked neomycin resistance cassette in reverse transcriptional orientation to the *Igh* locus, located immediately 5’ to the rearranged VDJ_H_^NIMP23^ variable region and its associated promoter (Figure 1—figure supplement 1D). Electroporation of C57BL/6N-derived PRX embryonic stem cells with the targeting construct and selection of homologous recombinant clones was performed using standard techniques by PolyGene AG (Switzerland). One targeted ES clone was used for production of chimeric mice using standard techniques at the Biological Research Facilities of the MRC National Institute for Medical Research, London, UK. Male chimeric mice were crossed to C57BL/6J females and progeny carrying the *Igh*^NIMP23neo^ allele were crossed to PC3Cre mice (O'Gorman et al., 1997) to delete the neo^r^ gene in the germline and generate mice carrying the *Igh*^NIMP23^ allele (Figure 1—figure supplement 1D). The *Igh*^NIMP23/+^ strain was maintained by backcrossing for at least 10 generations to C57BL/6J mice.

### Mixed bone marrow chimeras

Femurs and tibias were excised from female mice and cleaned of flesh using forceps and scalpel, and BM was obtained by flushing out with IMDM supplemented with 2 mM L-glutamine, 0.5 mM sodium pyruvate, 100 U penicillin, 100 mg streptomycin, 6 mM Hepes buffer, and 50 mM 2-ME (Gibco, Invitrogen), using a syringe with a needle. Thereafter, single BM cell suspensions were obtained by mashing through a 70 μm filter mesh, further sieved through 40 μm filter mesh and washed once. Live cells were resuspended in sodium chloride solution 0.9% (Sigma) at 4 × 10^6^ cells/200 μl. *Rag2^-/-^*.C57BL/6.SJL-*Ptprc^a^* mice were sub-lethally irradiated (5Gy) using a [137Cs] source and reconstituted less than 24 hr after irradiation by i.v. injections of a 10% *Igh*^NIMP23/+^:90% C57BL/6.SJL-*Ptprc^a^* combination of donor BM cells. Recipient mice were maintained on acidified drinking water and analyzed for reconstitution after 6–8 weeks.

### *Plasmodium chabaudi* infection

*Plasmodium chabaudi chabaudi AS* was transmitted by *Anopheles stephensi* mosquitoes, strain SD500, as described elsewhere (Spence et al., 2012). Briefly, C57BL/6J mice were injected i.p. with 10^5^ *P. chabaudi*-infected red blood cells and used to feed mosquitos two weeks after the injection. Two weeks after mosquito feeding/infection, each experimental mouse was exposed to 20 infected mosquitos for 30 min. Blood parasitemia in infected experimental mice was routinely monitored by thin blood smears.

### Immunizations

Mice were immunized i.p. with a combination of 100 μg of MSP1_21_ (Quin and Langhorne, 2001) and 50 μl of Titermax Gold emulsion (Sigma), or a combination of 50 μg of MSP1_21_ and 50 μg of R848 (Invivogen).

### Flow cytometry and cell sorting

Spleens, lymph nodes and bone marrows were dissected and single cell suspensions were obtained by mashing the organs through a 70 μm filter mesh in HBSS, 6 mM Hepes buffer (Gibco, Invitrogen). After removal of red blood cells from spleens and bone marrows by treatment with lysing buffer (Sigma), the remaining cells were resuspended in complete Iscove's Modified Dulbecco's Medium [IMDM supplemented with 10% FBS Serum Gold (PAA Laboratories, GE Healthcare), 2 mM L-glutamine, 0.5 mM sodium pyruvate, 100U penicillin, 100 mg streptomycin, 6 mM Hepes buffer, and 50 mM 2-ME (all from Gibco, Invitrogen)] and viable cells were counted using trypan blue (Sigma) exclusion and a hemocytometer. Cells were then resuspended in PBS and incubated with APC- or PE-labelled MSP1_21_ fluorescent probes and/or different combinations of fluorochrome-conjugated antibodies (key resources table), and either acquired after two washes with PBS, or fixed with 2% paraformaldehyde and stored in staining buffer at 4°C until acquisition.

The APC and PE MSP1_21_ fluorescent probes were produced as previously described (Krishnamurty et al., 2016; Taylor et al., 2012). Briefly, purified MSP1_21_ (Quin and Langhorne, 2001) was biotinylated using an EZ-link Sulfo-NHS-LC- Biotinylation kit (Thermo Fisher Scientic) using a 1:1 ratio of biotin to protein, and loaded onto Streptavidin-APC conjugated or Phycolink Streptavidin-R-PE conjugated (ProZyme) in a 6:1 ratio of MSP1_21_:Streptavidin-fluorochrome.

Cell sorting was performed on a MoFlo XDP (Beckman Coulter) or a BD FACSAria Fusion (BD Biosciences) and the target cell populations were directly dispensed into TRIreagent (Ambion) and stored at −80°C until RNA isolation. Purity checks were routinely performed for all assays by sorting aliquots of cells into PBS containing 2% FCS and reacquiring them on the cell sorter.

Dead cells were routinely excluded from the analysis by staining with LIVE/DEAD Fixable Aqua or Blue stain (Invitrogen). Singlets were selected based on FCS-A vs FCS-H and further based on SSC-A vs SSC-H. ‘Fluorescence minus one’ (FMO) controls were routinely used to verify correct compensation and to set the thresholds for positive/negative events. Analysis was performed with FlowJo software version 9.6 or higher (Tree Star).

PhenoGraph and *t*-distributed stochastic neighbor embedding (t-SNE) were combined to analyze multiparameter flow cytometry data using the Cytofkit package (Chen et al., 2016). t-SNE renders high-dimensional single-cell data based on similarities into only two dimensions, and thus helps visualize multiparameter data (van der Maaten, 2008). PhenoGraph (Levine et al., 2015) allows partitioning of high-dimensional single-cell data into phenotypically coherent subpopulations (i.e. clusters). The relatedness of the cell clusters identified by PhenoGraph was inferred using Isomap (Cytofkit package), in which related clusters/subsets can be visualised close to each other.

### RNA isolation, sequencing and data analysis

Total RNA from 1−5 × 10^4^ cells sorted into TRIreagent (Ambion) was isolated using the Ribopure kit (Ambion). Concentration of purified RNA was determined by Qubit fluorometric quantitation using the HS assay kit (ThermoFisher Scientific), and the quality analyzed with a 2100 Bioanalyzer (Agilent). Samples with a RIN score above 8.50 were used for the next steps. cDNA was generated from total RNA with the SMART-Seq v4 Ultra Low Input RNA Kit (Takara Bio USA). Next-generation sequencing libraries were produced with the Ovation Ultralow System V2 (Nugen), and run as PE100 on a HiSeq 4000 sequencer (Illumina). GEO accession: GSE115155.

For bioinformatics analysis, paired-end sequence reads were adapter and quality trimmed using cutadapt v1.9.1 (Martin, 2011) with the following non-default settings: ‘-a AGATCGGAAGAGC -A AGATCGGAAGAGC --minimum-length 30 -q 20,20’. Gene-level abundance estimates were generated from the trimmed reads using RSEM v1.2.31 (Li and Dewey, 2011) running STAR v2.5.1b (Dobin et al., 2013) with default settings, aligned against the *Mus musculus* Ensembl release 89 transcriptome (mm10). All further analysis was conducted using the DESeq2 (Love et al., 2014) package from Bioconductor v3.5 run in R v3.4.0. The expected counts were imported and rounded to integers to generate a counts matrix. Differential expression between phenotype groups was assessed using the DESeq function with default settings. In the case of comparisons of different MSP1_21_-specific B cell subsets obtained from the same experimental mouse, an additional mouse factor was added to the design formula to accommodate the paired nature of the data. Significance was thresholded using an FDR ≤ 0.01. PCA analysis was conducted using DESeq's plot PCA function with the regularized log (rlog) transformed count data. Heat maps were generated using the regularized log (rlog) transformed count data, scaled per gene using a z-score. Mouse homologues to genes previously associated with human AMB were selected (Supplementary file 1), and those showing significant differential expression on MSP1_21_-specific AMB were used to produce separate heat maps split by functional annotation (Figure 3). The GSEA pre-ranked function from the Broad's Gene Set Enrichment Analysis (GSEA) (Subramanian et al., 2005) suite was used to assess significant enrichment of MSigDB's C2 Reactome gene sets associated with differential expression between cell types. The function was run using a list of genes ranked for differential expression using DESeq2's Wald test statistic with default settings except for: collapse dataset to gene = false enrichment statistic = classic

### Statistical analysis

Statistical analysis was performed using Mann Whitney U test, Kruskal-Wallis test followed by Dunn's multiple comparisons test, or Two-Way ANOVA followed by Dunnett's multiple comparisons test on Prism software version 6 (GraphPad). p<0.05 was accepted as a statistically significant difference.

## Additional files

Supplementary file 1. Mouse homologues to human genes previously described to be either up (↑) or down (↓) regulated in human atypical memory B cells (AMB).

Supplementary file 2. List of top 50 Reactome gene sets yielding the highest normalized enrichment score (NES) by GSEA.

Transparent reporting form

### Data availability

RNAseq transcriptome data have been deposited in GEO under accession code GSE115155.

The following dataset was generated:

Pérez-MazliahDGardnerPJSchweighofferEMcLaughlinSHoskingCTumwineIDavisRSPotocnikATybulewiczVLanghorneJ2018Plasmodium-specific atypical memory B cells are short-lived activated B cellsNCBI Gene Expression OmnibusGSE11515510.7554/eLife.39800PMC624255330387712

## References

[bib1] Achtman AH, Stephens R, Cadman ET, Harrison V, Langhorne J (2007). Malaria-specific antibody responses and parasite persistence after infection of mice with Plasmodium chabaudi chabaudi. Parasite Immunology.

[bib2] Anderson SM, Tomayko MM, Ahuja A, Haberman AM, Shlomchik MJ (2007). New markers for murine memory B cells that define mutated and unmutated subsets. The Journal of Experimental Medicine.

[bib3] Ayieko C, Maue AC, Jura WG, Noland GS, Ayodo G, Rochford R, John CC (2013). Changes in B Cell Populations and Merozoite Surface Protein-1-Specific Memory B Cell Responses after Prolonged Absence of Detectable P. falciparum Infection. PLoS ONE.

[bib4] Barnett BE, Staupe RP, Odorizzi PM, Palko O, Tomov VT, Mahan AE, Gunn B, Chen D, Paley MA, Alter G, Reiner SL, Lauer GM, Teijaro JR, Wherry EJ (2016). Cutting Edge: B Cell-Intrinsic T-bet Expression Is Required To Control Chronic Viral Infection. The Journal of Immunology.

[bib5] Baumgarth N, Tung JW, Herzenberg LA (2005). Inherent specificities in natural antibodies: a key to immune defense against pathogen invasion. Springer Seminars in Immunopathology.

[bib6] Baumgarth N (2011). The double life of a B-1 cell: self-reactivity selects for protective effector functions. Nature Reviews Immunology.

[bib7] Bhattacharya D, Cheah MT, Franco CB, Hosen N, Pin CL, Sha WC, Weissman IL (2007). Transcriptional profiling of antigen-dependent murine B cell differentiation and memory formation. The Journal of Immunology.

[bib8] Boyle DB, Newbold CI, Smith CC, Brown KN (1982). Monoclonal antibodies that protect in vivo against Plasmodium chabaudi recognize a 250,000-dalton parasite polypeptide. Infection and Immunity.

[bib9] Brugat T, Reid AJ, Lin J, Cunningham D, Tumwine I, Kushinga G, McLaughlin S, Spence P, Böhme U, Sanders M, Conteh S, Bushell E, Metcalf T, Billker O, Duffy PE, Newbold C, Berriman M, Langhorne J (2017). Antibody-independent mechanisms regulate the establishment of chronic Plasmodium infection. Nature Microbiology.

[bib10] Carpio VH, Opata MM, Montañez ME, Banerjee PP, Dent AL, Stephens R (2015). IFN-γ and IL-21 Double Producing T Cells Are Bcl6-Independent and Survive into the Memory Phase in Plasmodium chabaudi Infection. PloS ONE.

[bib11] Charles ED, Brunetti C, Marukian S, Ritola KD, Talal AH, Marks K, Jacobson IM, Rice CM, Dustin LB (2011). Clonal B cells in patients with hepatitis C virus-associated mixed cryoglobulinemia contain an expanded anergic CD21low B-cell subset. Blood.

[bib12] Chen H, Lau MC, Wong MT, Newell EW, Poidinger M, Chen J (2016). Cytofkit: A Bioconductor Package for an Integrated Mass Cytometry Data Analysis Pipeline. PLoS Computational Biology.

[bib13] Clark AG, Chen S, Zhang H, Brady GF, Ungewitter EK, Bradley JK, Sackey FN, Foster MH (2007). Multifunctional regulators of cell growth are differentially expressed in anergic murine B cells. Molecular Immunology.

[bib14] Davis RS, Stephan RP, Chen CC, Dennis G, Cooper MD (2004). Differential B cell expression of mouse Fc receptor homologs. International Immunology.

[bib15] Davis RS (2007). Fc receptor-like molecules. Annual Review of Immunology.

[bib16] de Bree GJ, Wheatley AK, Lynch RM, Prabhakaran M, Grijsen ML, Prins JM, Schmidt SD, Koup RA, Mascola JR, McDermott AB (2017). Longitudinal dynamics of the HIV-specific B cell response during intermittent treatment of primary HIV infection. PloS ONE.

[bib17] Dobin A, Davis CA, Schlesinger F, Drenkow J, Zaleski C, Jha S, Batut P, Chaisson M, Gingeras TR (2013). STAR: ultrafast universal RNA-seq aligner. Bioinformatics.

[bib18] Dorfman JR, Bejon P, Ndungu FM, Langhorne J, Kortok MM, Lowe BS, Mwangi TW, Williams TN, Marsh K (2005). B cell memory to 3 Plasmodium falciparum blood-stage antigens in a malaria-endemic area. The Journal of Infectious Diseases.

[bib19] Ehrhardt GR, Davis RS, Hsu JT, Leu CM, Ehrhardt A, Cooper MD (2003). The inhibitory potential of Fc receptor homolog 4 on memory B cells. PNAS.

[bib20] Ehrhardt GR, Hsu JT, Gartland L, Leu CM, Zhang S, Davis RS, Cooper MD (2005). Expression of the immunoregulatory molecule FcRH4 defines a distinctive tissue-based population of memory B cells. The Journal of Experimental Medicine.

[bib21] Enghard P, Humrich JY, Chu VT, Grussie E, Hiepe F, Burmester GR, Radbruch A, Berek C, Riemekasten G (2010). Class switching and consecutive loss of dsDNA-reactive B1a B cells from the peritoneal cavity during murine lupus development. European Journal of Immunology.

[bib22] Fabregat A, Jupe S, Matthews L, Sidiropoulos K, Gillespie M, Garapati P, Haw R, Jassal B, Korninger F, May B, Milacic M, Roca CD, Rothfels K, Sevilla C, Shamovsky V, Shorser S, Varusai T, Viteri G, Weiser J, Wu G, Stein L, Hermjakob H, D’Eustachio P (2018). The Reactome Pathway Knowledgebase. Nucleic Acids Research.

[bib23] Garraud O, Borhis G, Badr G, Degrelle S, Pozzetto B, Cognasse F, Richard Y (2012). Revisiting the B-cell compartment in mouse and humans: more than one B-cell subset exists in the marginal zone and beyond. BMC Immunology.

[bib24] Illingworth J, Butler NS, Roetynck S, Mwacharo J, Pierce SK, Bejon P, Crompton PD, Marsh K, Ndungu FM (2013). Chronic exposure to Plasmodium falciparum is associated with phenotypic evidence of B and T cell exhaustion. The Journal of Immunology.

[bib25] Johnson G, Wu TT (2001). Kabat Database and its applications: future directions. Nucleic Acids Research.

[bib26] Kabashima K, Haynes NM, Xu Y, Nutt SL, Allende ML, Proia RL, Cyster JG (2006). Plasma cell S1P1 expression determines secondary lymphoid organ retention versus bone marrow tropism. The Journal of Experimental Medicine.

[bib27] Kallies A, Hasbold J, Fairfax K, Pridans C, Emslie D, McKenzie BS, Lew AM, Corcoran LM, Hodgkin PD, Tarlinton DM, Nutt SL (2007). Initiation of plasma-cell differentiation is independent of the transcription factor Blimp-1. Immunity.

[bib28] Kardava L, Moir S, Wang W, Ho J, Buckner CM, Posada JG, O'Shea MA, Roby G, Chen J, Sohn HW, Chun TW, Pierce SK, Fauci AS (2011). Attenuation of HIV-associated human B cell exhaustion by siRNA downregulation of inhibitory receptors. Journal of Clinical Investigation.

[bib29] Kardava L, Moir S, Shah N, Wang W, Wilson R, Buckner CM, Santich BH, Kim LJ, Spurlin EE, Nelson AK, Wheatley AK, Harvey CJ, McDermott AB, Wucherpfennig KW, Chun TW, Tsang JS, Li Y, Fauci AS (2014). Abnormal B cell memory subsets dominate HIV-specific responses in infected individuals. Journal of Clinical Investigation.

[bib30] Knox JJ, Buggert M, Kardava L, Seaton KE, Eller MA, Canaday DH, Robb ML, Ostrowski MA, Deeks SG, Slifka MK, Tomaras GD, Moir S, Moody MA, Betts MR (2017a). T-bet+ B cells are induced by human viral infections and dominate the HIV gp140 response. JCI Insight.

[bib31] Knox JJ, Kaplan DE, Betts MR (2017b). T-bet-expressing B cells during HIV and HCV infections. Cellular Immunology.

[bib32] Krishnamurty AT, Thouvenel CD, Portugal S, Keitany GJ, Kim KS, Holder A, Crompton PD, Rawlings DJ, Pepper M (2016). Somatically Hypermutated Plasmodium-Specific IgM. Immunity.

[bib33] Lalor PA, Nossal GJ, Sanderson RD, McHeyzer-Williams MG (1992). Functional and molecular characterization of single, (4-hydroxy-3-nitrophenyl)acetyl (NP)-specific, IgG1+ B cells from antibody-secreting and memory B cell pathways in the C57BL/6 immune response to NP. European Journal of Immunology.

[bib34] Levine JH, Simonds EF, Bendall SC, Davis KL, Amir el-AD, Tadmor MD, Litvin O, Fienberg HG, Jager A, Zunder ER, Finck R, Gedman AL, Radtke I, Downing JR, Pe'er D, Nolan GP (2015). Data-Driven Phenotypic Dissection of AML Reveals Progenitor-like Cells that Correlate with Prognosis. Cell.

[bib35] Li B, Dewey CN (2011). RSEM: accurate transcript quantification from RNA-Seq data with or without a reference genome. BMC Bioinformatics.

[bib36] Li H, Borrego F, Nagata S, Tolnay M (2016). Fc receptor-like 5 expression distinguishes two distinct subsets of human circulating tissue-like memory b cells. The Journal of Immunology.

[bib37] Love MI, Huber W, Anders S (2014). Moderated estimation of fold change and dispersion for RNA-seq data with DESeq2. Genome Biology.

[bib38] Martin M (2011). Cutadapt removes adapter sequences from high-throughput sequencing reads. EMBnet.journal.

[bib39] Moir S, Ho J, Malaspina A, Wang W, DiPoto AC, O'Shea MA, Roby G, Kottilil S, Arthos J, Proschan MA, Chun TW, Fauci AS (2008). Evidence for HIV-associated B cell exhaustion in a dysfunctional memory B cell compartment in HIV-infected viremic individuals. The Journal of Experimental Medicine.

[bib40] Muellenbeck MF, Ueberheide B, Amulic B, Epp A, Fenyo D, Busse CE, Esen M, Theisen M, Mordmüller B, Wardemann H (2013). Atypical and classical memory B cells produce Plasmodium falciparum neutralizing antibodies. The Journal of Experimental Medicine.

[bib41] Naradikian MS, Hao Y, Cancro MP (2016a). Age-associated B cells: key mediators of both protective and autoreactive humoral responses. Immunological Reviews.

[bib42] Naradikian MS, Myles A, Beiting DP, Roberts KJ, Dawson L, Herati RS, Bengsch B, Linderman SL, Stelekati E, Spolski R, Wherry EJ, Hunter C, Hensley SE, Leonard WJ, Cancro MP (2016b). Cutting Edge: IL-4, IL-21, and IFN-γ Interact To Govern T-bet and CD11c Expression in TLR-Activated B Cells. The Journal of Immunology.

[bib43] Ndungu FM, Cadman ET, Coulcher J, Nduati E, Couper E, Macdonald DW, Ng D, Langhorne J (2009). Functional memory B cells and long-lived plasma cells are generated after a single Plasmodium chabaudi infection in mice. PLoS Pathogens.

[bib44] Ndungu FM, Olotu A, Mwacharo J, Nyonda M, Apfeld J, Mramba LK, Fegan GW, Bejon P, Marsh K (2012). Memory B cells are a more reliable archive for historical antimalarial responses than plasma antibodies in no-longer exposed children. PNAS.

[bib45] Ndungu FM, Lundblom K, Rono J, Illingworth J, Eriksson S, Färnert A (2013). Long-lived Plasmodium falciparum specific memory B cells in naturally exposed Swedish travelers. European Journal of Immunology.

[bib46] O'Gorman S, Dagenais NA, Qian M, Marchuk Y (1997). Protamine-Cre recombinase transgenes efficiently recombine target sequences in the male germ line of mice, but not in embryonic stem cells. PNAS.

[bib47] Obeng-Adjei N, Portugal S, Tran TM, Yazew TB, Skinner J, Li S, Jain A, Felgner PL, Doumbo OK, Kayentao K, Ongoiba A, Traore B, Crompton PD, Yeste A, Mascanfroni ID, Nadeau M, Burns EJ, Tukpah A-M, Santiago A, Wu C, Patel B, Kumar D, Quintana FJ (2015). Circulating th1-cell-type tfh cells that exhibit impaired b cell help are preferentially activated during acute malaria in children. Cell Reports.

[bib48] Obeng-Adjei N, Portugal S, Holla P, Li S, Sohn H, Ambegaonkar A, Skinner J, Bowyer G, Doumbo OK, Traore B, Pierce SK, Crompton PD (2017). Malaria-induced interferon-γ drives the expansion of Tbethi atypical memory B cells. PLoS Pathogens.

[bib49] Pérez-Mazliah D, Ng DH, Freitas do Rosário AP, McLaughlin S, Mastelic-Gavillet B, Sodenkamp J, Kushinga G, Langhorne J (2015). Disruption of IL-21 signaling affects T cell-B cell interactions and abrogates protective humoral immunity to malaria. PLoS Pathogens.

[bib50] Phan TG, Amesbury M, Gardam S, Crosbie J, Hasbold J, Hodgkin PD, Basten A, Brink R (2003). B cell receptor-independent stimuli trigger immunoglobulin (Ig) class switch recombination and production of IgG autoantibodies by anergic self-reactive B cells. The Journal of Experimental Medicine.

[bib51] Pillai S, Cariappa A, Moran ST (2005). Marginal zone B cells. Annual Review of Immunology.

[bib52] Portugal S, Pierce SK, Crompton PD (2013). Young lives lost as B cells falter: what we are learning about antibody responses in malaria. The Journal of Immunology.

[bib53] Portugal S, Tipton CM, Sohn H, Kone Y, Wang J, Li S, Skinner J, Virtaneva K, Sturdevant DE, Porcella SF, Doumbo OK, Doumbo S, Kayentao K, Ongoiba A, Traore B, Sanz I, Pierce SK, Crompton PD (2015). Malaria-associated atypical memory B cells exhibit markedly reduced B cell receptor signaling and effector function. eLife.

[bib54] Portugal S, Obeng-Adjei N, Moir S, Crompton PD, Pierce SK (2017). Atypical memory B cells in human chronic infectious diseases: An interim report. Cellular Immunology.

[bib55] Quin SJ, Langhorne J (2001). Different regions of the malaria merozoite surface protein 1 of Plasmodium chabaudi elicit distinct T-cell and antibody isotype responses. Infection and Immunity.

[bib56] Ridderstad A, Tarlinton DM (1998). Kinetics of establishing the memory B cell population as revealed by CD38 expression. Journal of Immunology.

[bib57] Rivera-Correa J, Guthmiller JJ, Vijay R, Fernandez-Arias C, Pardo-Ruge MA, Gonzalez S, Butler NS, Rodriguez A (2017). Plasmodium DNA-mediated TLR9 activation of T-bet^+^ B cells contributes to autoimmune anaemia during malaria. Nature Communications.

[bib58] Rubtsov AV, Marrack P, Rubtsova K (2017). T-bet expressing B cells - Novel target for autoimmune therapies?. Cellular Immunology.

[bib59] Rubtsova K, Rubtsov AV, van Dyk LF, Kappler JW, Marrack P (2013). T-box transcription factor T-bet, a key player in a unique type of B-cell activation essential for effective viral clearance. PNAS.

[bib60] Russell Knode LM, Naradikian MS, Myles A, Scholz JL, Hao Y, Liu D, Ford ML, Tobias JW, Cancro MP, Gearhart PJ (2017). Age-associated b cells express a diverse repertoire of v_h_ and vκ genes wit_h_ somatic hypermutation. The Journal of Immunology.

[bib61] Sen J, Rosenberg N, Burakoff SJ (1990). Expression and ontogeny of CD2 on murine B cells. Journal of Immunology.

[bib62] Spence PJ, Jarra W, Lévy P, Nahrendorf W, Langhorne J (2012). Mosquito transmission of the rodent malaria parasite Plasmodium chabaudi. Malaria Journal.

[bib63] Spence PJ, Jarra W, Lévy P, Reid AJ, Chappell L, Brugat T, Sanders M, Berriman M, Langhorne J (2013). Vector transmission regulates immune control of Plasmodium virulence. Nature.

[bib64] Subramanian A, Tamayo P, Mootha VK, Mukherjee S, Ebert BL, Gillette MA, Paulovich A, Pomeroy SL, Golub TR, Lander ES, Mesirov JP (2005). Gene set enrichment analysis: a knowledge-based approach for interpreting genome-wide expression profiles. PNAS.

[bib65] Sullivan RT, Kim CC, Fontana MF, Feeney ME, Jagannathan P, Boyle MJ, Drakeley CJ, Ssewanyana I, Nankya F, Mayanja-Kizza H, Dorsey G, Greenhouse B (2015). FCRL5 Delineates Functionally Impaired Memory B Cells Associated with Plasmodium falciparum Exposure. PLoS Pathogens.

[bib66] Sullivan RT, Ssewanyana I, Wamala S, Nankya F, Jagannathan P, Tappero JW, Mayanja-Kizza H, Muhindo MK, Arinaitwe E, Kamya M, Dorsey G, Feeney ME, Riley EM, Drakeley CJ, Greenhouse B (2016). B cell sub-types following acute malaria and associations with clinical immunity. Malaria Journal.

[bib67] Taki S, Meiering M, Rajewsky K (1993). Targeted insertion of a variable region gene into the immunoglobulin heavy chain locus. Science.

[bib68] Taylor JJ, Martinez RJ, Titcombe PJ, Barsness LO, Thomas SR, Zhang N, Katzman SD, Jenkins MK, Mueller DL, Martinez RJ, Mueller DL (2012). Deletion and anergy of polyclonal B cells specific for ubiquitous membrane-bound self-antigen. The Journal of experimental medicine.

[bib69] Tomayko MM, Steinel NC, Anderson SM, Shlomchik MJ (2010). Cutting edge: Hierarchy of maturity of murine memory B cell subsets. The Journal of Immunology.

[bib70] van der Maaten L (2008). Visualizing Data using t-SNE. Journal of Machine Learning Research.

[bib71] Weiss GE, Crompton PD, Li S, Walsh LA, Moir S, Traore B, Kayentao K, Ongoiba A, Doumbo OK, Pierce SK (2009). Atypical memory B cells are greatly expanded in individuals living in a malaria-endemic area. The Journal of Immunology.

[bib72] Weiss GE, Traore B, Kayentao K, Ongoiba A, Doumbo S, Doumtabe D, Kone Y, Dia S, Guindo A, Traore A, Huang CY, Miura K, Mircetic M, Li S, Baughman A, Narum DL, Miller LH, Doumbo OK, Pierce SK, Crompton PD (2010). The Plasmodium falciparum-specific human memory B cell compartment expands gradually with repeated malaria infections. PLoS Pathogens.

[bib73] Weiss GE, Clark EH, Li S, Traore B, Kayentao K, Ongoiba A, Hernandez JN, Doumbo OK, Pierce SK, Branch OH, Crompton PD (2011). A positive correlation between atypical memory B cells and Plasmodium falciparum transmission intensity in cross-sectional studies in Peru and Mali. PLoS ONE.

[bib74] Wipasa J, Suphavilai C, Okell LC, Cook J, Corran PH, Thaikla K, Liewsaree W, Riley EM, Hafalla JC (2010). Long-lived antibody and B Cell memory responses to the human malaria parasites, Plasmodium falciparum and Plasmodium vivax. PLoS Pathogens.

[bib75] Won WJ, Foote JB, Odom MR, Pan J, Kearney JF, Davis RS (2006). Fc receptor homolog 3 is a novel immunoregulatory marker of marginal zone and B1 B cells. The Journal of Immunology.

[bib76] Young F, Ardman B, Shinkai Y, Lansford R, Blackwell TK, Mendelsohn M, Rolink A, Melchers F, Alt FW (1994). Influence of immunoglobulin heavy- and light-chain expression on B-cell differentiation. Genes & Development.

[bib77] Zhu Z, Li R, Li H, Zhou T, Davis RS (2013). FCRL5 exerts binary and compartment-specific influence on innate-like B-cell receptor signaling. PNAS.

[bib78] Zouali M (2011). Marginal zone B-cells. A Gatekeeper of Innate Immunity.

[bib79] Zuccarino-Catania GV, Sadanand S, Weisel FJ, Tomayko MM, Meng H, Kleinstein SH, Good-Jacobson KL, Shlomchik MJ (2014). CD80 and PD-L2 define functionally distinct memory B cell subsets that are independent of antibody isotype. Nature Immunology.

